# Healthcare and education networks interaction as an indicator of social services stability following natural disasters

**DOI:** 10.1038/s41598-021-81130-w

**Published:** 2021-01-18

**Authors:** Emad M. Hassan, Hussam Mahmoud

**Affiliations:** Department of Civil and Environmental Engineering, Colorado State University, Fort Collins, CO USA

**Keywords:** Health care, Engineering, Mathematics and computing

## Abstract

Healthcare and education systems have been identified by various national and international organizations as the main pillars of communities’ stability. Understanding the correlation between these main social services institutions is critical to determining the tipping point of communities following natural disasters. Despite being defined as social services stability indicators, to date, no studies have been conducted to determine the level of interdependence between schools and hospitals and their collective influence on their recoveries following extreme events. In this study, we devise an agent-based model to investigate the complex interaction between healthcare and education networks and their overall recovery, while considering other physical, social, and economic factors. We employ comprehensive models to simulate the functional processes within each facility and to optimize their recovery trajectories after earthquake occurrence. The results highlight significant interdependencies between hospitals and schools, including direct and indirect relationships, suggesting the need for collective coupling of their recovery to achieve full functionality of either of the two systems following natural disasters. Recognizing this high level of interdependence, we then establish a social services stability index, which can be used by policymakers and community leaders to quantify the impact of healthcare and education services on community resilience and social services stability.

## Introduction

Maintaining social services stability following natural disasters is critical for sustainable cities and communities. Achieving proper functionality level of healthcare and education facilities, in particular, is key to bring normalcy back to communities and reduce the potential for population outmigration, which commonly occurs as an outcome of shortage in their services^1^. Therefore, it is not a surprise that various national and international organizations recognize the importance of education and healthcare systems to communities’ stability. For example, the United Nations listed quality education and good health and well-being as two of its 17 sustainable development goals for the year 2030^2^. Similarly, requirements for substantial advancements in these two systems are part of UNICEF's New Strategic Plan^3^ for the years 2018–2021. This international recognition of the importance of good education and healthcare systems has also been noted in the United States (U.S.) by various leading scientific organizations, including the National Academy of Engineering^4^, the National Institute of Standard and Technology^5^, and the National Science Foundation^6^. More specifically, the National Institute for Standards and Technology included schools and hospitals among the essential institutions for providing social services stability within a community^7^.

Historical events have shown both schools and hospitals to be vulnerable to extreme natural disasters^8–10^. For instance, the 1989 Loma Prieta earthquake damaged two hospitals^11^ and resulted in severe damage to three schools^12^, the 1995 Kobe earthquake collapsed four hospitals^13^ and damaged approximately 4,500 schools^14^, and the 2008 Sichuan earthquake caused the collapse of many hospitals and schools^15^. Earthquake damages also resulted in total casualties of 35,000, 3757, and 10,000 from the Loma Prieta, PDD Kobe, and Sichuan earthquakes, respectively^13^. These casualties created a medical surge for the already affected healthcare facilities^11^, which significantly impacted the offered medical services in terms of accessibility and effectiveness. Damage to school buildings can result in death and injuries to staff and students^15^, increase in post-traumatic stress for schoolchildren^16^, and complete halt of the education service due to school closure^1^.

Because of their significant potential role in enhancing health and long-term economic mobility in a community, disturbance to healthcare and education networks can seriously impact the welfare of the society^17^. Policymakers have recognized that achieving good health is tied to eliminating poverty and providing proper education^17,18^. It has also been shown that the health condition of individuals can be an essential factor in their success at school and in the workplace^17,18^. Recognizing this interaction, many schools have partnered with hospitals in the U.S. to address mental health issues and other health concerns for students^19^. Recent efforts in the U.S. have been geared towards aligning education and health metrics through the Healthy Schools campaign at a state level or through a partnership between the Healthy Schools Project partnered with Trust for America’s Health and others. Emphasis has been placed on incorporating health metrics into education accountability systems and on integrating education metrics into the healthcare system^19^. Natural disasters have a major impact on schools and hospitals and can significantly impact their interaction, particularly for highly interdependent systems, which has not been quantified in previous studies. A higher degree of interdependency between the built environment can increase community vulnerability and complicate recovery after any disruption^20^. The National Institute for Standards and Technology, in their Community Resilience Planning Guide^7^ highlighted the importance of the dependencies within and among the so-called social institutions. Interdependencies can be classified into functional, physical, budgetary, social, and economical^21^. Different approaches have been utilized to model infrastructure interdependency, including empirical, agent-based, system dynamics, economic theories, and network analysis^21^. Identifying the degree of interdependency between the built environment is challenging as it requires comprehensive models to simulate all possible processes and decisions made within each of these infrastructures and their impact on each other. There is a shortage of analytical models that can be used to simulate large social systems; let alone the interdependency between these systems. Previous studies on quantifying interedependencies between hospitals and schools were either empirical^20^, statistical^22^, or described as a theory with no quantification^23^. The dependency between healthcare and education services can be direct or indirect^24^. Direct interdependency can only capture the simple relationship between the investigated services. On the other hand, the indirect interdependency pertains to quantifying the more complicated relationship between (a) the investigated service providers, (b) each provider and their supporting infrastructure that form the community, and (c) the sub-components within each provider. Capturing such complex interaction requires more detailed models that can mimic the disruptive events within each facility as well as the impact of each facility on the other.

Agent‐based models are common for simulating complex systems and networks, and they have been applied in many fields, including epidemiology, business, and social science^25^. An agent comprises a set of autonomous decision-making entities that can be individuals, groups, or systems^26^. These entities have a set of characteristics and rules that allows them to interact, learn, and adapt. Modeling agents’ behaviors and interactions can be conducted using self-contained algorithms or logical operations formalized by equations^27^. Agent-based modeling is a robust method for investigating systems’ behavior, study the relationships among their dynamic components, and present a natural description of complicated systems. Furthermore, it offers sufficient level of flexibility to accommodate different levels of system complexity in which features such as aggregation of agents, agent sub-components, and different levels of descriptions for agents can be represented^27^. However, these models have several limitations, including the uncertainty associated with their expected results^28^.

The functionality of social institutions, such as healthcare and education systems, can be measured using the quantity and quality of the offered services at each facility^29–32^. To model these complicated systems, various components need to be considered. The main components of the healthcare system are healthcare providers, patients, regulators, payers, and suppliers^33,34^, while the main components of the education system are education providers, students, parents, administration, community, regulators, and suppliers^35^. Each of these providers, with hospitals or schools as the central components, comprises of various sub-components. Different frameworks have been introduced to simulate healthcare and education systems' functionality, independently, after natural disasters. However, most previous studies were limited to a single facility^36,37^, neglected the interdependency between the investigated facilities and other community lifelines^38,39^, stopped short of including the system dynamics in terms of capacity and demand^40,41^, or only considered the quantity part of the offered services^31,41^. To overcome some of these limitations, however, recent studies focused on developing new physical-based frameworks, based on components’ realistic behavior, to model healthcare^34^ and education systems^35^. In these frameworks, the healthcare service functionality was measured as a combination of the available staffed beds as an indicator of the healthcare service quantity and patient waiting time and patient treatment time as indicators of the healthcare service quality^34^. On the other hand, education service functionality was quantified in terms of the school enrollment capacity as an indicator of the education service quantity and a combination of teacher, classroom, and school qualities as indicators of the education service quality^35^.

Once functiality is determined, resilience of a system can be estmatied. There are amble definitions of resilience that span different fields and disciplines, starting from the early definition of ecological resilience by Holling^42^ to a recent definition of infrastructure resilience by the White House^43^ as noted in the Presidential Policy Directive-21 (PPD-21). For example, in the PPD-21, resilience is defined as the ability to prepare for and adapt to changing conditions and withstand and recover rapidly from disruptions. The European Commission (EC), on the other hand, noted the importance of considering resilience at multiple scales while bearing in mind sustainable developments. That is resilience is defined by the EC as the ability of an individual, a household, a community, a country, or a region to withstand, cope, adapt, and quickly recover from stresses and shocks such as violence, conflict, drought, and other natural disasters without compromising long-term development^44^. Various efforts^45,46^ have been carried out worldwide with focus on improving the built environment and/or enhancing human capacity to ensure rapid recovery as per the resilience definitions. From a quantitive perspective, various studies have been conducted to develop mathematical models^47,48^ and tools^49,50^ to measure resilience by determining the cumulative functional loss for the built environment^47^, economic^51^, and social^52^ parameters. A wide spectrum of single and/or compounded indices can be used to measure community resilience including such as employment rate, household income, education attainment, and hospital capacity, among others^53^.

Despite recent advances in quantifying cummultiave functional loss and resilience of healthcare and education systems, to date, studies on their interaction, their collective effect on their respective recovery, and the stability of the social services of communities are lacking. Quantifying the interaction, especially between these social institutions, is critical for community resilience analysis^7,20,48^. Their compounded role in societies is essential for building robust communities^17^, informing public policies^5,6^, and influencing social indices^54,55^. Here, we devise an agent-based model to investigate the interaction between healthcare and education systems as well as their impact on resilience and social services stability of communities after natural disasters. The model is structured using a socio-technical approach that is based on guidelines and case studies for real communities after disasters. The developed model is then tested on a mid-size virtual community to develop an estimate for the level of interdependency between each healthcare and education facility. The results are then used to construct a social services stability index, which can be used to quantify the impact of healthcare and education services on community resilience and social stability.

## Model application

**Figure 1:**
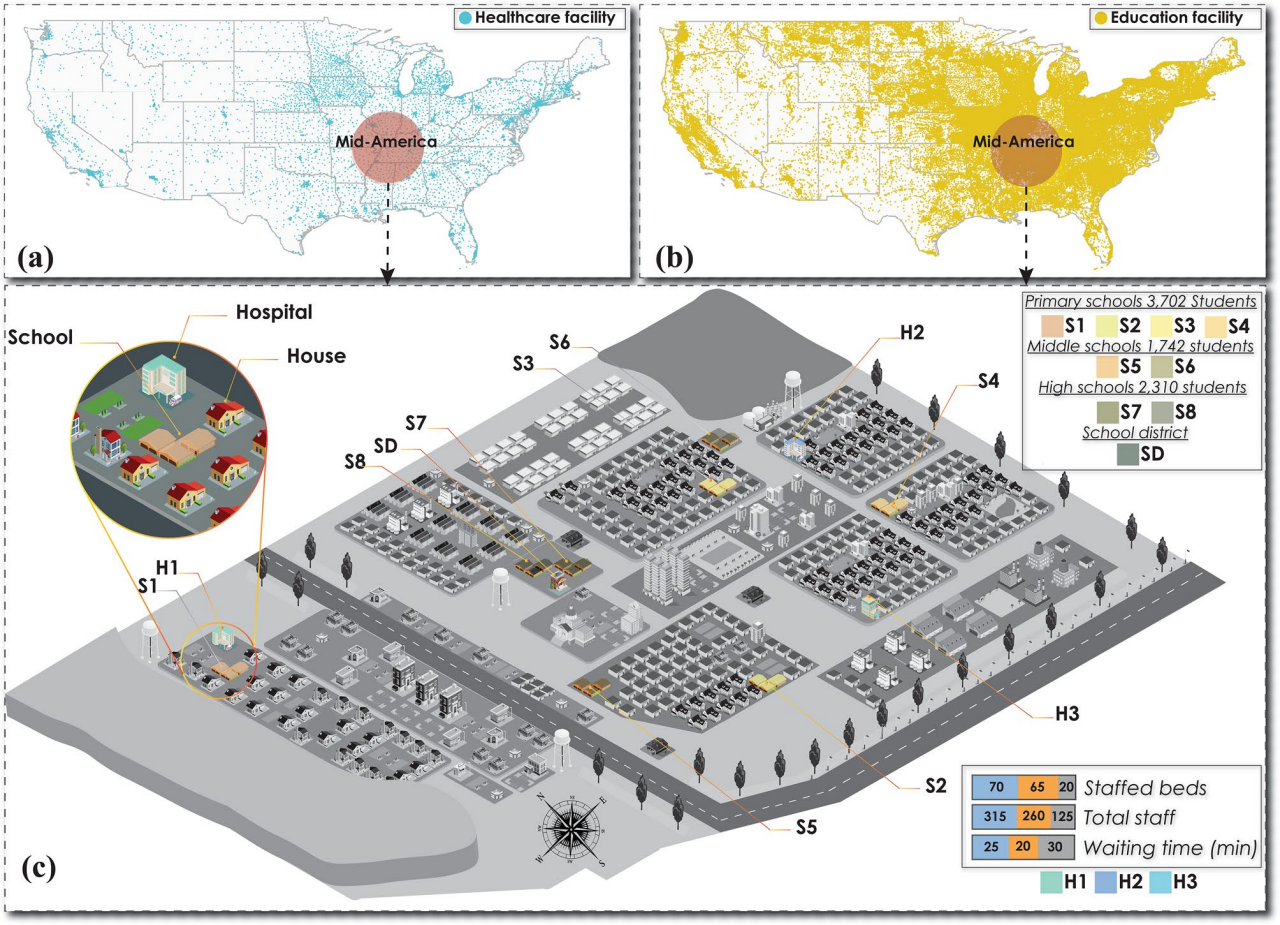
Healthcare and education facilities distribution in the U.S. in 2020: (**a**) healthcare, (**b**) education, and (**c**) the investigated virtual community, which is a mid-size community in the Mid-America region with 50,000 total population served by three hospitals and eight schools. Hospital capacity and school enrollment are listed in figure (**c**).

A virtual community, shown in Fig. 1, was built to represent a typical middle-sized community in the mid-America region^56^. Three hospitals with 70, 65, and 20 total staffed beds with 315, 260, and 125 staff, respectively, are considered as the healthcare service providers^34^. There are eight schools distributed as four primary, two middle, and two high schools as the education service providers. The total number of students is 925, 871, and 1155 with the corresponding number of staff of 97, 91, and 161 for the primary, middle, and high school, respectively^35^.

Hospitals and schools’ relevant data are obtained to construct the agent-based model as summarized in Table S1 in the Supplementary Information (SI) for the investigated community. These data include the functionality of different sub-components, available mitigation strategies, and resources at each facility^34,35,56^. Hospitals and school’s total functionalities are modeled (Section S1 and S2 in the SI, respectively). Patients are distributed to the healthcare facilities during normal operation using decision-making heuristics as part of the introduced agent-based model as outlined in Fig. S4 and Section S3 in the SI. Interaction among the hospitals is outlined in Fig. S5 and discussed in Section S3 of the SI. These frameworks are utilized to distribute patients and simulate the transfer of resources between healthcare facilities based on the community’s demographic data^56^. Furthermore, schools’ enrollment (Fig. S6 and Section S3 in the SI) is defined using data related to the school zones, number and location of each school’s staff and schoolchildren in the investigated community^56^. Optimal recovery of hospitals and schools and the community’s infrastructure after an earthquake scenario are modeled using stochastic analysis coupled with dynamic optimization (Section S4 in the SI).

## Results

### Interdependence between healthcare and education facilities

**Figure 2:**
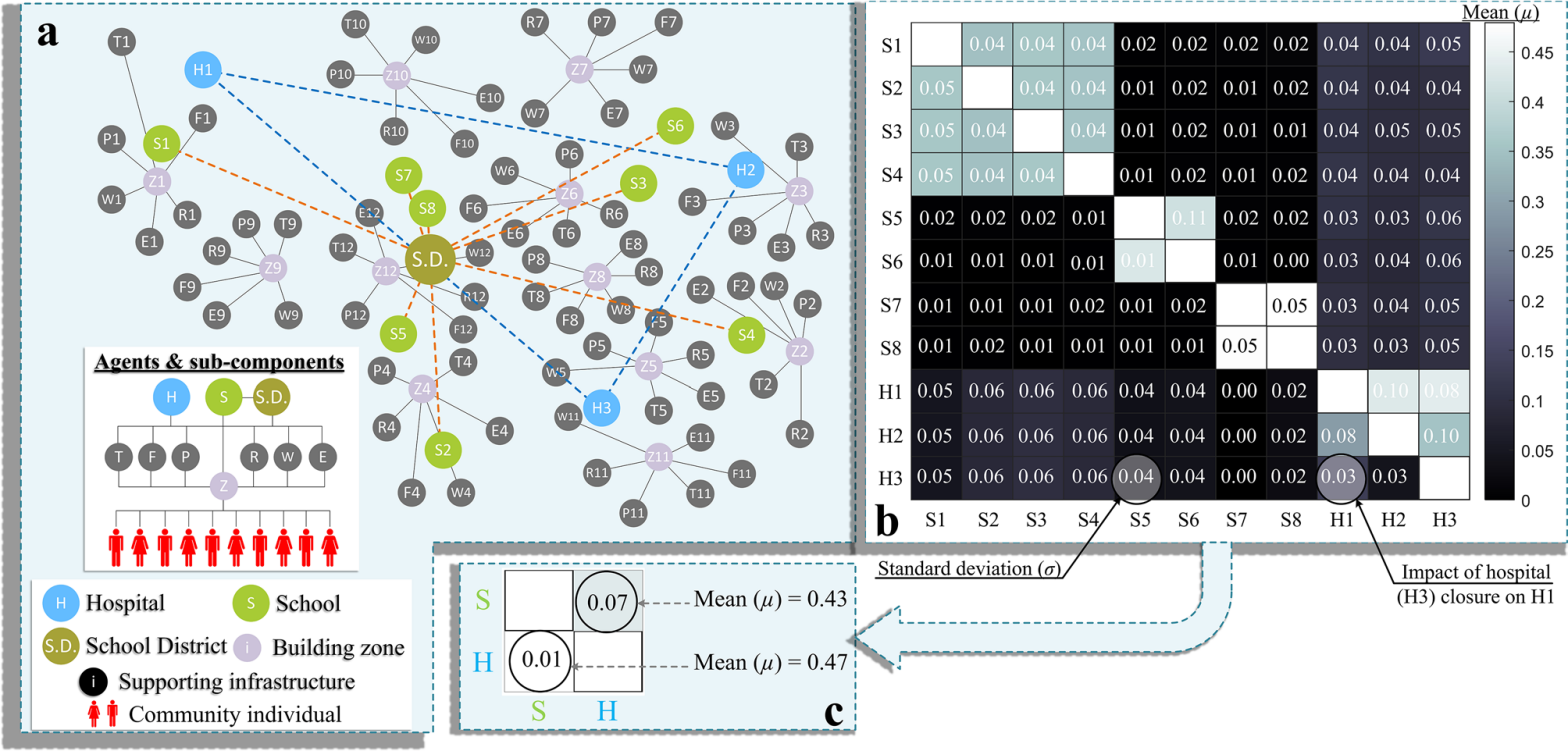
(**a**) Components of the complex network representing the agent-based model of the investigated community, (**b**) interdependency matrix between each healthcare and education facility, and (**c**) interdependency between the healthcare and education systems.

To quantify the interdependence between the healthcare and education facilities, an agent-based model comprising two main agents (representing hospitals and schools) as well as supporting agents (representing their supporting infrastructure) and sub-agents (representing individuals in the community) is devised. The interaction among the agents, supporting agents, and sub-agents is described by the network in Fig. 2a. Further details on agent type, attributes, and possible decisions can be found in Material and methods, Table S1, and Sections S1–S3 in the SI. In this study, interdependency is quantified, among single facilities as well as between the healthcare and education systems, using the Leontief-based model^57^. The model captures the impact of total functionality drops at either a single facility or the whole system, which can be healthcare or education, on the other facilities or other systems. The uncertainty associated with the residents of staff, patients, and schoolchildren within the community, as well as those associated with quality functionality of hospitals and schools’ sub-components, are also included in the analysis. Monte-Carlo simulation and statistical distributions are used to develop a relationship, ***N***(*µ, σ*), between the functionality of each investigated facility.

For the relationship between single facilities (Fig. 2b), strong dependence is noted between hospitals as well as schools with the same grades. This is a result of redistributing patients and students impacted by increased waiting time at hospitals and reaching class capacity at schools. The impact of any single school closure on hospitals is shown to range from 9 to 12% and is influenced by the shortage in hospital staff where their children either changed their school, shifted to homeschooling, or missed school all together. The impact of any single hospital closure on the schools can be up to 10% and is associated with staff shortage and students’ chronic absenteeism. This impact is mainly caused by a reduction in healthcare services provided to the school staff and students, the long waiting time in emergency departments, and early discharge of staff and students before receiving the appropriate treatment during one school year (180 days). The hospitalization data for school staff or students are based on U.S. hospitalization statistics^58^ and is a function of the patient’s age. The relationship between the healthcare and education system is also investigated as shown in Fig. 2c, which demonstrates the impact of the lack of functionality of the entire system on the other. The analysis shows a higher degree of interdependency between the two systems where a complete drop of healthcare functionality can reduce that of the education by 47% and increase students’ chronic absenteeism by 22.4%. On the contrary, a complete drop of education functionality is expected to reduce that of the healthcare by 43%. This level of dependency is much higher than what was conceived in previous empirical^20^, statistical^22^, or theoretical^23^ studies.

### Resilience of the healthcare and education systems

**Figure 3:**
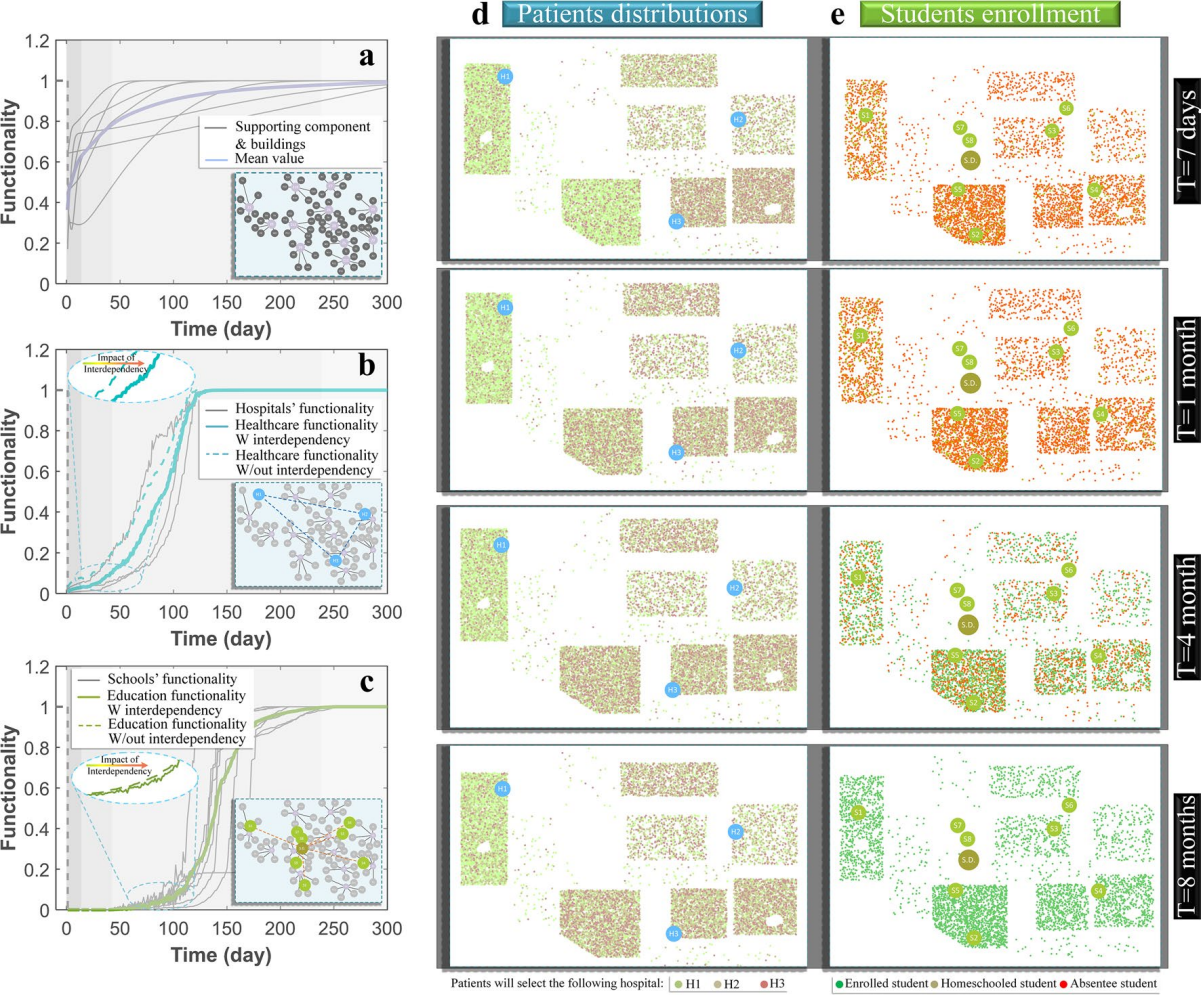
The functionality of (**a**) supporting infrastructure, (**b**) healthcare system, and (**c**) education system, in addition to the change of (**d**) patient distributions, and (**e**) student enrollment status after the earthquake disaster.

To investigate the resilience of the main social institutions in the tested community, the resilience quantification framework, which is outlined in Fig. S7 of the SI is utilized by subjecting the community to an earthquake scenario with *M*_*w*_ of 7.9 and an epicentral distance of approximately 10 km from the Southwest of the community. The earthquake-associated damage to the healthcare and education facilities, as well as their supporting agents and the interaction topology, is modeled after Hassan and Mahmoud^34^ and Hassan et al.^35^ (see Sections S1–S2 in the SI). Direct losses, including the earthquake casualties related to the investigated facilities’ staff and users, are calculated separately using Hazus MH 2.1^59^ (see Fig. S8 in the SI). Staff casualties directly impact the functionality of hospitals and schools and users’ casualties increase the demand on hospitals and chronic absenteeism in schools (see Section S3 in the SI). The recovery of the supporting infrastructure and buildings in the investigated community (Fig. 3a) is estimated using data from ATC-13^60^ (see Fig. S9 and Fig. S10 in the SI for damage and recovery of supporting infrastructure, respectively). Recovery of the hospital and school agents, shown in Fig. 3b,c, are estimated using a semi-Markov-chain stochastic analysis while optimal repair resources allocation is determined using dynamic optimization to maximize the total number of staffed beds for hospitals and the enrollment capacity of the schools (see Section S4 in the SI). Optimal decisions are modeled to utilize available resources and maintain the offered services by each facility (see Material and methods).

The supporting infrastructure is shown to recover 80% of its functionality in about one month (Fig. 3a), which indicates that the earthquake damage is slight and the impact on the community’s physical infrastructure is minor. Full recovery for the healthcare system is achieved within four months (Fig. 3b), while for the education system, more than eight months are needed for full recovery (Fig. 3c). Comparing the estimated functionality when considering and ignoring the relationship between the healthcare and education systems highlights the fact that delaying the restoration of one service can negatively impact the other (Fig. 3b,c). Schools’ closure reduces the healthcare system functionality until all schoolchildren of the hospital staff are back to schools. The hospitals’ drop in functionality reduces the student outcomes by increasing the absenteeism of staff and students. Patient demand on healthcare facilities is a dynamic process, as shown in Fig. 3d. The most probable hospital for each patient is shown to be sensitive to the functionality of the transportation network as well as each facility. Education service for most of the students is discontinued for more than three months, which represents a full semester in the schools’ academic calendar (Fig. 3e). Schools’ enrollment is affected by the schools’ buildings’ safety. However, providing backup systems and backup spaces accelerates the schools’ opening time after the earthquake (see Material and methods). Homeschooling, which can be used by families for the first few months after the earthquake, is a significant option for continuing the education of their children, but it is not available for all families in the investigated community either because of student’s guardian restrictions or fulfillment of different federal and state regulations^61^.

### Community social services stability index

Here, we establish a new notion of a so-called social services stability index (*SSSI*), which, on a scale from zero to one, is meant to indicate how social services are stable for the community’s residents as influenced by the availability of school and hospital services. The *SSSI* is calculated by integrating healthcare and education services while considering the need for each individual in the community to each of the investigated services. The mathematical integration of the two services is shown in detail in Material and methods. The *SSSI* is expected to drop directly after the earthquake but then slowly rise during the recovery stage.

**Figure 4:**
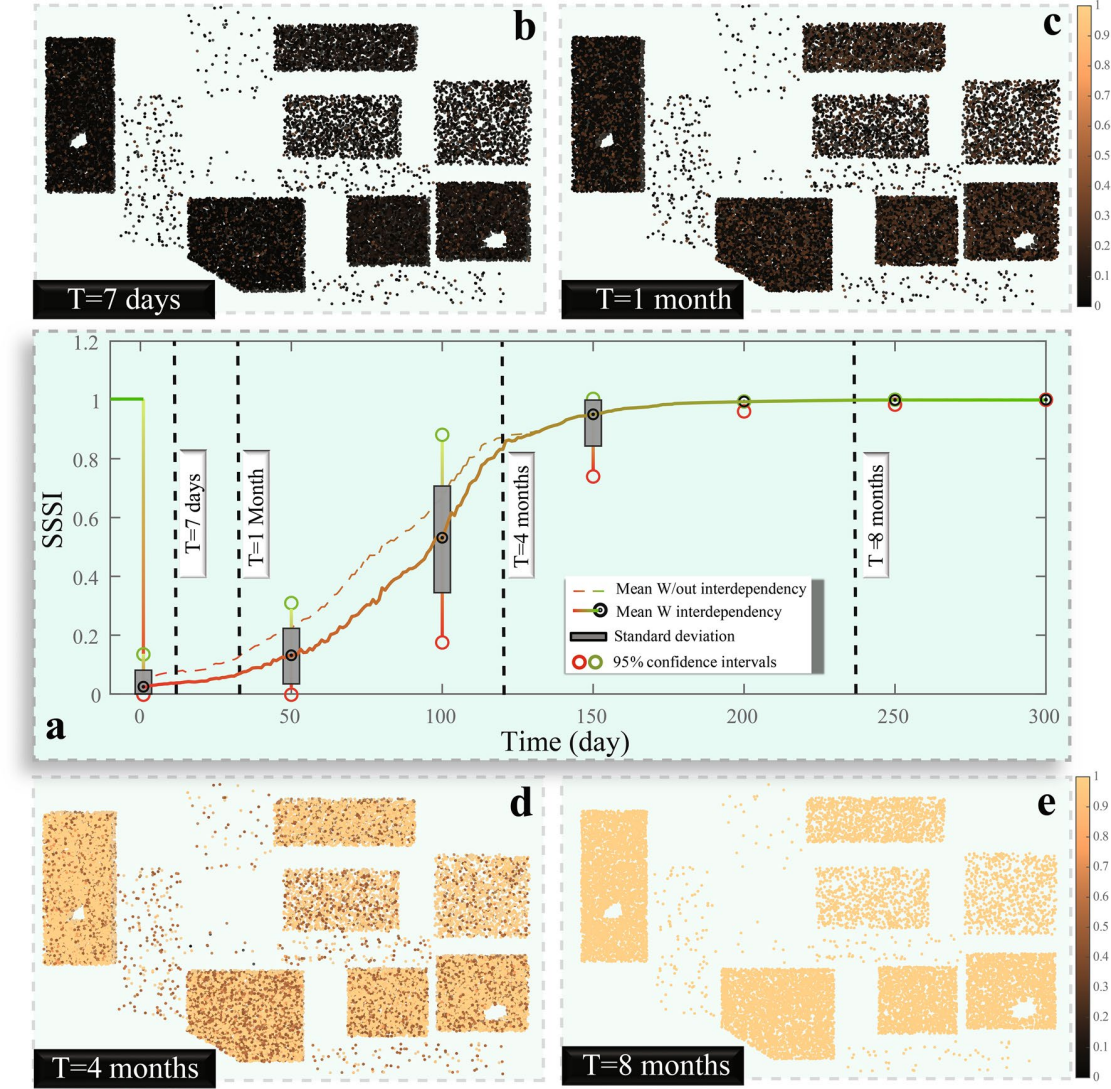
(**a**) Recovery of the *SSSI* over time, and the spatial distribution of the *SSSI* after (**b**) 1 week, (**c**) 1 month, (**d**) 4 months, and (**e**) eight months.

During the first week after the earthquake, and due to the increase in patient demand and reduced available staffed beds in hospitals, a significant increase in the patients’ waiting time in the crowded emergency departments is recorded (Fig. 4). Despite the effectiveness of the dynamic triage criteria, reduction in treatment time and patient early discharge (a common practice by hospitals to increase the survival rate of the patients with high severities), lowers the patient outcome for less severe cases (see Material and methods). This situation is also combined with the closure to all the schools in the community, which results in a substantial drop (more than 96%) in the *SSSI* for most individuals of the community. Driven by the recovery of the healthcare facilities and reduction of the patient demand, the *SSSI* reaches 12% of its original value prior to the earthquake in a month and a half. By the end of four months, the *SSSI* exceeds 83%, which is driven by the full recovery of the healthcare functionality and opening of most of the schools. Finally, eight months after the disaster, the *SSSI* is close to its initial value, and the community returns to normalcy. Variation in healthcare and education services for individuals in the community is significant during the recovery time and produces disparities in the accessibility of the main services.

## Discussion

The primary objective of this study was to develop a generalized approach to investigate the interdependency between complex networks of healthcare and education systems and their impact on community resilience and social services stability after earthquake events. Comprehensive frameworks used to quantify the seismic resilience of healthcare and education systems were coupled with a novel agent-based model comprising of system’s agents, main agents, and sub-agents. We found a strong relationship between the two networks and showed that their level of interdependency can change over time after any disruptive events. We revealed that interdependencies between schools and healthcare facilities could go beyond employees and direct users of these services and encompass other individuals with minor needs for these services because of their social relationships with others who directly interface with these services. We also uncovered that the facility size and interaction topology, being healthcare or education, can play a critical role in the level of interdependency among different facilities.

From the results, we also observe that earthquakes can be devastating for mid-size communities despite the minor damage to the community’s built environment, including the physical infrastructure. We note that the community’s social services stability is sensitive even to minor disturbances. Therefore, more attention should be given to quantifying the resilience of healthcare facilities and schools in communities. Providing alternatives for healthcare and education services providers is critical for the community’s social stability. These alternatives include patients and resources transfer between hospitals, homeschooling for school students, and backup systems and backup spaces for hospitals and schools, which can have a major impact on the quantity functionality of the investigated services. We show that the change of community social services stability is dynamic and more sensitive to any disturbance compared with other community’s built environment. Furthermore, a holistic and comprehensive definition of the *SSSI* requires consideration of the interaction between different service providers.

**Figure 5:**
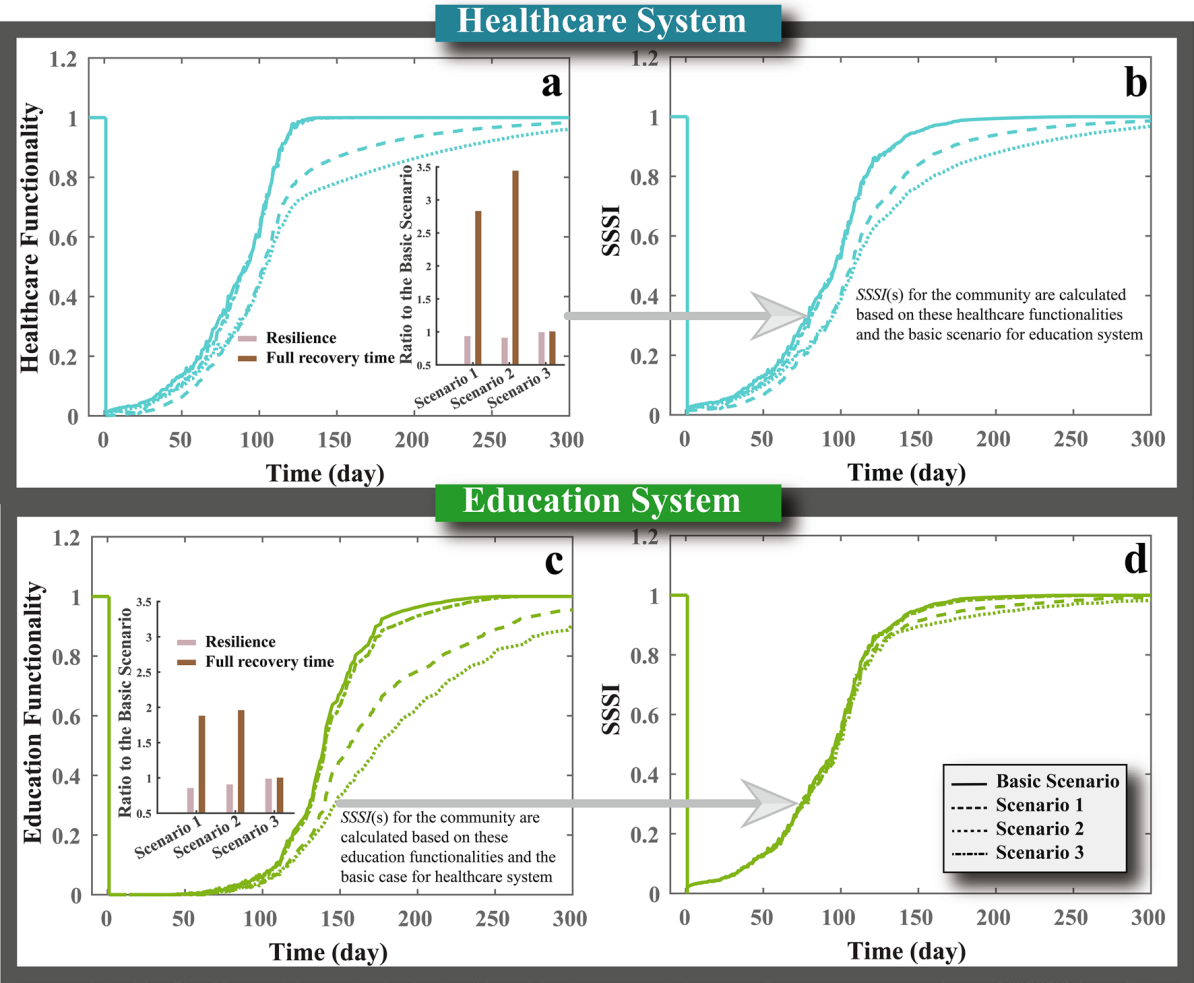
Impact of failing to apply different mitigation strategies on (**a**) the total functionality of healthcare system, (**b**) community’s *SSSI*, (**c**) the total functionality of education system, and (**d**) community’s *SSSI*. The Basic Scenario case refers to the case when all the mitigation strategies applied, Scenario 1, represents the case when the no mitigation for staff, Scenario 2, denotes the case when no mitigation strategies for utilities and space are applied, and Scenario 3, indicate the case when no mitigation strategies are implemented.

Different mitigation strategies are assumed to be applied by hospitals and schools to overcome the shortage of staff, space, and supplies after the earthquake. These practical mitigation strategies are considered based on guidelines, recommendations, and lessons learned after previous natural disaster events. For instance, utilizing alternative staff, transferring staff from other facilities, and assigning additional working hours for existing staff at hospitals and schools are recommended to close the staff shortage gap^8,34,35,62–65^. Providing hospitals and schools with backup systems and backup spaces are also recommended mitigation strategies that could provide hospitalization and education services without the need for permanent utilities or space^34,35,62,66–71^. In addition, using alternative supplies, optimizing the supply usage, and transferring supplies between different facilities can be significant in reducing supply shortage, limiting the services provided by these facilities^34,35,63,71,72^. To further reflect on the sensitivity of healthcare and education systems to disturbances to their relevant socio-physical parameters, we evaluate four different cases to observe the impact of each case on recovery trajectories and the computed *SSSI* for the community, as shown in Fig. 5. The four cases are Basic Scenario (when all the mitigation strategies are applied), Scenario 1 (when no strategies are used to manage staff shortage), Scenario 2 (when no strategies are utilized to overcome utilities outage and space damage), and Scenario 3 (when no strategies are applied to close the gap in supplies). We found that failing to apply these mitigation strategies will reduce the functionality, delay the facilities opening and recovery time, and impact the community *SSSI*. Failing to manage and replace staff can diminish the healthcare system functionality for more than 21 days after the earthquake and delay the attainment of full recovery for more than 240 days. While its impacts on the education system will not be notable immediately after schools’ reopening, lack of proper management and staff replacement will impact the quality of the education service and delays the full recovery of the service for more than 221 days. The backup systems and spaces are the most critical for hospitals and schools in the short and long terms. While backup systems are utilized to reopen facilities earlier, the backup spaces are essential for these facilities to continue providing services when the repair process of building components is underway. Failing to provide the required backup systems and spaces will delay the full recovery of the healthcare and education systems by 320 and 241 days, respectively. Failing to maintain the supply availability will significantly reduce the healthcare system functionality immediately after the earthquake; however, its impact will be minor in the long-term. While its impact on education system functionality will be insignificant, lack of supplies can delay schools reopening for 21 days.

While the introduced models can provide substantial insight into how the healthcare and education systems interact and their impact on communities, there are different limitations that should be reflected upon when using the proposed approach. First, although the focus of the study was on healthcare and education systems and their interaction in the aftermath of extreme events, the resilience of other socio-economic sectors, such as food suppliers and retailers, are also important for maintaining an adequate level of social services for communities. Furthermore, other resilience goals (governance stability or population stability, etc.), measured by different metrics, might be more relevant for some communities and can be integrated with the recovery of healthcare and education systems for a more complete assessment of resilience and the *SSSI* (see Material and methods). Although it was not included in this study, it is acknowledged that some communities might choose to return to a higher level of functionality by incorporating the concept of building back better so that they are more resilient against future events^73,74^. Finally, other methods can be used to quantify the parameters in the presented models, and a detailed examination of the sensitivity of the calculated parameters to the different models is needed. Despite the mentioned limitations, the presented frameworks and modeling approach can also be applicable to evaluate the impact of other hazards and diseases on the healthcare and education systems.

## Material and methods

### Agent-based model attributes

**Figure 6:**
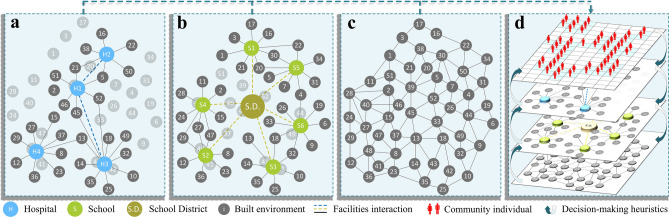
Components of the agent-based model: (**a**) healthcare system including its supporting infrastructure, (**b**) education system comprising of schools, school district as a main administrative component, and the supporting infrastructure, (**c**) the community built environment containing infrastructure, building, and suppliers, and (**d**) interaction between community individuals (sub-agents), healthcare and education facilities (main agents), and the built environment (supporting agents) in the decision-making heuristics stage.

The introduced model (Fig. 6) comprises of (a) main agents (healthcare and education facilities and all their sub-components), supporting agents (community’s buildings and infrastructure that supports the functionality of the main agents), and sub-agents (community individuals); (b) decision-making heuristics and learning rules; (c) an interaction topology including buildings, supportive infrastructure, and suppliers for both the healthcare and education facilities; and (d) an environment. The entire framework is structured as a multi-layered agent-based model, system, main agents, and sub-agents, in which the system represents the entire networks of hospitals or schools. Each of these systems is defined by a group of main agents (either healthcare or education facilities); each is supported by supporting agents representing the built environment including water, power, transportation, telecommunication, wastewater, natural gas, and buildings as well as a group of medical and non-medical suppliers. Furthermore, each of these main agents is dependent on sub-agents that represent all community individuals and includes different staff classes as operators and regulators as well as patients, students, or students’ guardians as expected service receptors.

### Agents description and interaction topology

The healthcare system is modeled through the interaction of various sub-components, including personnel, *ST*, main utilities, *U*, space, *SP*, and supplies, *SU*, (Fig. S1 in the SI). *ST* is categorized into physicians, nurses, supporting staff, and alternative staff, and are all modeled as sub-agents. *U* refers to the essential services provided to the healthcare system from the community’s infrastructure, which includes water, power, transportation, telecommunication, wastewater, and drinking water, and are all framed as supporting agents. *SP* comprises structural, non-structural, and contents sub-components as well as building accessibility, which are all simulated as main agents. *SU* reflects the daily necessities and supplies for the medical facilities such as oxygen, surgical, *RX*, fuel, and food, among others and is simulated as a supporting agent. The availability of these sub-components is assembled using the previously developed complex healthcare network interaction model^34^ to estimate the functionality healthcare system. The probability of availability of sub-component, *i*, is integrated with the total number of sub-components, *n*, $P_{B} = {\prod\nolimits_{i = 1}^{n}} {P_{i} }$, to define the hospital’s ability to receive and provide patients, which is expressed in terms of the available staffed beds, *B*, and is conditioned on the required service being available. To provide a full description of the functionality of the healthcare system, *Q*_*H*_, the quantity of the service, *Q*_*V*_, is combined with the accessibility*, S*_*A*_*,* and effectiveness*, S*_*E*_*,* of this service as an indication of the service quality, *Q*_*S*_, as $Q_{H} \left( t \right) = Q_{V} \left( t \right)^{\alpha_{V} } Q_{S} \left( t \right)^{\alpha_{S} }$; where *α*_*V*_ and *α*_*V*_ are weighting factors for service quantity and quality, respectively. *Q*_*V*_ is calculated based on *B* and its type, being an emergency or inpatient bed, as shown in Fig. S1.

The education system is simulated, similar to the healthcare network model, through the interaction of different sub-components including staff, *ST*, main utilities, *U*, physical components, *SP*, and supplies, *U*, (Fig. S2 in the SI). *ST* is subclassified into teachers, supporting staff, administrative staff, and volunteers, which are all modeled as sub-agents. *U* is provided by the schools’ supporting infrastructure and comprises of water, power, transportation, telecommunication, wastewater, and drinking water, all of which are framed as supporting agents. School’s *SP* includes structural, non-structural, and contents as well as building accessibility, which are all simulated as main agents. *SU* includes books, fuel, and food, among others, and is simulated as a supporting agent. These sub-components are used to calculate the functionality of the education system^35^, including schools’ enrollment capacity, *N*_*i*_, which is an indicator of the school’s quantity functionality, *S*_*V*_, in each grade, *i*. The expected value of the school’s quantity functionality, *E*[*S*_*V*_], is calculated for the whole school as follows $E\left[ {S_{V} } \right] = \sum\nolimits_{i = i}^{I_{g} } {\frac{1}{N_{i} }\sum\nolimits_{n = 1}^{{N_{i} }} {P_{s,i}^{n} } }$; where *I*_*g*_ and *N*_*i*_ are the total numbers of grades and enrollment capacity at grade *i*, respectively. In addition, the quality of the education service provided by schools, *S*_*S*_, is also measured using indicators related to teacher, classroom, and school quality. Teacher quality is measured by teacher assignment, *T*_*a*_, and experience, *T*_*e*_. Classroom quality is measured by class size, *C*_*s*_, and technology, *C*_*t*_. School quality is measured by leadership, *S*_*l*_, and professional community, *S*_*pc*_. These indicators are combined with the school quantity functionality to formulate the school’s total functionality, *S*_*S*_, as $S_{S} \left( t \right) = S_{V} \left( t \right)^{\alpha_{V} } S_{S} \left( t \right)^{\alpha_{S} }$.

The supporting agents are the community’s built environment, including infrastructure and buildings that support the functionality of the investigated facilities, as shown in Fig. 6a,b. These agents are part of the community physical components shown in Fig. 6c. Buildings, including where the staff of the investigated facilities reside, are modeled as supporting agents to estimate the availability of staff in each facility. They are also used to locate staff, patients, and students, as well as the travel time for these individuals to the investigated facilities. Damage and functionality of these buildings are used to estimate the casualties after the earthquake and the expected number of the population that needs to be relocated. Utilities that refer to the supporting infrastructure for hospitals and schools are modeled as supporting agents. The functionality of the infrastructure denotes the availability of the service provided by them at the investigated facilities. The supporting agents also include the suppliers for healthcare and education facilities. The functionality of these suppliers is utilized to estimate the full availability of the investigated facilities’ supplies. The interdependency between these components is modeled using previous studies^20^, and their interaction with the investigated facilities is simulated in the functionality models for hospitals and schools as discussed previously. These interactions between community individuals (sub-agents), healthcare and education facilities (main agents), and the built environment (supporting agents) are depicted in Fig. 6d.

In addition to the main and supporting agents discussed previously, all community individuals are modeled as sub-agents. These sub-agents are classified based on their relation to the main agents, as hospitals or schools’ staff, patients, students, or/and student guardians. The spatial and temporal relationships among these sub-agents as well as between the sub-agents and both the main and supporting agents are modeled. For instance, the relationship between school staff and schoolchildren is modeled where any family can be directly impacted by the shortage in healthcare or education services if one of the family members works at one of these service providers. The indirect effect is also captured if a family member is a user of these facilities. Furthermore, these sub-agents are modeled to be impacted by the functionality of the supporting agents, including utilities and buildings. For example, if staff members of hospitals or schools have no housing or utilities, they will not be able to work since they must relocate. These sub-agents are also dynamically simulated such that during the investigated time frame the relation between these sub-agents and main and supporting agents can change. For example, staff can change their working facilities within the same system; patients can alter their most-probable hospitals; students can move to other schools.

### Decision-making heuristics

Decisions in the presented study can be made by main agents, sub-agents, or supporting agents. The sub-agents’ decisions directly impact the agents they are related to and the system formed by these agents and indirectly influence other systems as well (Table S1 in the SI). The decisions are made based on current functionality states, available resources, and alternatives to achieve a set of objectives that ensure services’ availability. To that end, a simulation model is developed to mimic the agent’s different functions and choices before, during, and after the event and to resolve problems resulting from any disturbances in the modeled agents. These judgments are simulated using algorithms that are structured based on regulations, previously reported situations, and case studies (Fig. S3 in the SI). The algorithms first locate the source of the functionality drop, *Γ*, which is defined as the least functional of all sub-components, as shown in Eq. 1. Then multi-objective optimization is used to find the optimal solution or in some cases a combination of solutions, *X*_Γ_*, among multiple *X*_Γ_* ∈ (x_*Γ*,*1*_, …, *x*_*Γ*_*,*_*n*_*)*, based on available resources, *Ψ*, to maximize the functionality, *F*, with minimal use of *Ψ* as follow:

1 $$\Gamma = min\left( {ST,SP,U,SU,\left\{ {S_{A} ,S_{E} or T_{a} ,T_{e} ,C_{c} ,C_{t} ,S_{l} ,S_{pc} } \right\}} \right)$$

2 $$\mathop {\max }\limits_{{\rm X}_{\Gamma }^{*} } F\left( {x_{\Gamma ,i} ,\mathop {min}\limits_{\Psi_{\Gamma }^{*} } (\Psi_{\Gamma ,i} )} \right) \forall i \in \left( {1, \ldots n} \right)$$

The algorithm considers different approaches to increase the capacity and functionality of the healthcare system and reduce the overwhelming demand for them. These approaches have been applied and proven effective in enhancing the operation of healthcare systems after disasters. The decisions made by the sub-agents and supporting agents are embedded in these approaches. For the sub-agents, the algorithm accommodates the use of alternative staff, accept staff transfer from other facilities, and assign additional working hours for existing staff, which have been noted as viable options in enhancing functionality^63^. Other approaches can also be implemented, such as reducing patient treatment time and discharging patients with minor severities. However, hospitals’ staff must cautiously apply these approaches as it can negatively affect the patients’ outcomes and reduce the effectiveness of the patients’ treatment. The selection between these approaches is subjected to resources availability. Different approaches can also be combined to maximize the hospital’s quantity functionality while considering other sub-components, *ST* = *max*(*U*, *SP*, *SU*). Similarly, for the supporting agents, hospital backup systems might be used in the case of utility shortage and backup spaces for the case of space damage. Accelerating the restoration process and shortening the recovery time can also assist in increasing the availability of utilities and space, which is modeled by optimizing the distribution and transfer of repair resources among the investigated facilities. To overcome the shortage in supplies, hospitals can find alternative suppliers, transfer supplies between hospitals, and optimize the supplies usage. These approaches can be combined to increase the supplies to the same level as other sub-components *SU* = *max*(*ST*, *U*, *SP*). One of the main components that impact hospital quality functionality, in terms of patient waiting time, is the expected number of patients, which is calculated using a patient-driven model (Fig. S4 in the SI) and is expected to increase after major disasters. To deal with the increase in patient numbers, *N*_*n*_, at a hospital, *n*, beyond the capacity, *B*_*n*_, the healthcare system can adopt dynamic triage criteria^75^, reduce patient treatment time^76^, employ early discharge for non-critical cases, and transfer patients to other facilities. Another approach that can be implemented to reduce the patient waiting time is to share resources between the healthcare facilities including ambulances as well as available staffed bed data in each facility^31^.

Unlike the healthcare facilities, which are mostly independently managed, public schools, which represent 90% of the U.S. schools, are centrally managed by the school district and are governed by school boards and superintendents. The administrative role played by these sectors is critical for the school system to adapt and enhance its performance. The presented algorithm for the school administration includes the decision made to (a) find alternatives for the impacted sub-components, (b) facilitate student admission and transfer, (c) close and reopen schools after disasters, and (d) monitor the quality of the service offered by each school. The role played by the sub-agents and supporting agents is modeled in each of these decisions. To find alternatives to close the gap in the school’s staff, schools coordinate with their school district to assign additional teaching loads to existing teachers, allocate volunteers for the community to substitute the supporting staff, accept staff transfer from other schools, and appoint temporary and part-time staff. The total number of required staff, *ST*_*i,req*_, at grade *i*, is calculated as $ST_{i,req} = N_{i} {/}R_{i}$, which is based on the number of students enrollment at this grade, *N*_*i*_, and the classroom capacity, *R*_*i*_. School administration can decide to increase the class’s capacity and, in some cases, apply the double sessions system in which students are divided into groups that attend at different times of the day. However, these decisions also have a higher impact on the students’ outcome, which is considered in the utilized algorithm. School administration can also offer alternatives for schools’ utilities by providing backup systems or, in limited cases, run schools without some utilities. The school districts can arrange with each school to provide backup or alternative spaces as a replacement for the original non-functional buildings. To provide essential supplies at each school, the school districts can find alternative suppliers and arrange supplies transfer between schools. One of the main responsibilities of school districts is student admission and transfer and the arrangement of student transportation. Because of natural disasters, school transportation can be impacted; therefore, the school districts can arrange different transportation methods for students, including public and private transportations. Decisions made to reopen the damaged schools after major disasters require safe and functional school space and approval of different entities, including the school district, the building and fire departments, the office of public safety, and the community, etc. The school district coordinates with each school to enhance the education service quality provided by each facility by setting clear criteria to replace any less experienced and unqualified staff to enhance teacher and school quality, appoint more staff to increase the teacher assignment, and apply previously mentioned approaches to reduce the class size and increase the availability of technology in the classroom.

### Environment

The environment component in the introduced agent-based model defines each component and sub-component location, including all individuals in the community. The environment is dynamic and changes with time to reflect disturbances and damages in all components of the community to allow updating of the travel time, patient distribution, schoolchildren admission, transfer of resources, and repair process after the disaster, etc.

### Healthcare and education demand modeling

#### Patient distribution

One of the main variables that impact the facility’s functionality, in the presented healthcare system model, is patient demand, which changes based on various socio-economic parameters. A Patient-driven algorithm is used in this study to identify these parameters and distribute the expected communities’ daily patients to the healthcare system facilities. This patient distribution algorithm utilizes the decisions made by the sub-agents to define the patient constraints parameters, including case criticality and insurance, and the reputation parameters such as media effect, previous positive experience, and brand name. The algorithm also considers the connection between the patient and the facility and includes transportation and travel time while accounting for decisions made by the main agents in terms of the facility’s ability to receive this patient based on waiting time and ability to treat the individual. A probability tree analysis is used to combine these parameters, and the most probable facility for each patient is then identified^34^ (Fig. S4 in the SI). After receiving the patients, hospitals can transfer some of them due to certain circumstances such as patient surge resulting in the capacity being exceeded, limited treatment ability for the patient case, or the need to evacuate the hospital. The patient transfer decision is made using the hospital interaction algorithm provided by Hassan and Mahmoud^34^ and is based on agreements between the main agents and sub-agents (sender facility, receiver facility, and the transferred patient) while considering the functionality of the supporting agents. This decision is made as a function of the patient’s case criticality, insurance type, the availability of a connection between the two facilities to transfer the patient and his/her records, and the ability of the receiver facility to accommodate this patient. Parameters such as the travel time between the two facilities, availability of transportation and telecommunication services, waiting time, and treatment ability at the receiver hospital are also considered in the decision process (Fig. S5 in the SI).

#### Student enrollment

In the introduced education system model, the student enrollment and transfer process are controlled by different variables such as the school’s enrollment capacity, predefined school zones, transportation options, and school of choice. While enrollment capacity is determined using the school functionality model, the school zone is defined based on the community's demographics and census data to distribute the schoolchildren to nearby schools. School of choice provides parents or guardians the option to select different schools for their schoolchildren. However, this option has more constraints related to school approval and transportation. Natural disasters can disturb the education system, which impacts student enrollment and transfer process. The presented algorithm can simulate the education system disturbance from natural disasters and adopt different approaches with the objective of returning the maximum number of students to schools and reduce chronic absenteeism^35^. School districts can also arrange other education alternatives for students, including homeschooling (Fig. S6 in the SI).

### Interdependency assessment

The introduced agent-based model is utilized to quantify the interdependency between healthcare and education on the system level and hospitals and schools on the agent level, which is an essential step towards understanding how the social institutions interact. These interdependencies are fundamental for the resilience and sustainability analysis, including but not limited to population dislocation and social vulnerability analysis. To estimate the functional interdependency between healthcare and education systems or agents, the Leontief-based model^57^ is utilized, as shown in Eq. 3.

3 $${\mathcal{F}}_{k} = \mathop \sum \limits_{j} \eta_{kj} {\mathcal{F}}_{j} + {\rm H}_{k} , \quad \forall k = 1,2, \ldots , n$$

where ℱ is the total functionality, η is the degree of interdependency, H is the inoperability risk of a system component, k.

### Recovery and stability

#### Restoration and resilience

Immediately after the disaster, the community will start the restoration process to bring the community back to normalcy. Decisions made on the system, agent, and sub-agent levels, during this stage are simulated in this study to investigate the impact of these decisions on the community recovery process and expected change of the individuals and community’s social services stability (Fig. S7 in the SI). The utilized approach divides the time after disaster into the assessment and planning stages to model the recovery stage. That is when each facility assesses the extent of damage and arrange the repair process. This stage is followed by the recovery stage, where the actual repair process starts, and the limited repair resources are distributed to achieve predefined goals of the community, which is achieving the same level of functionality prior to an event. Semi-Markov chain process coupled with dynamic optimization is utilized in this study to allocate the repair resources to the damaged facilities to estimate the optimal recovery path that attain the community objectives in terms of offering the maximum number of staffed beds in all the hospitals and enrollment capacity for students in all grades.

In this study, we utilized the PPD definition of resilience as noted in the Introduction. Community resilience performance goals can be divided into population stability, economic stability, social services stability, physical services stability, and governance stability^77^. Each one of these five goals can be measured by different resilience metrics. For example, population stability can be measured by the number of households dislocated, percent of the population remaining in the community, etc. We choose to focus on social services stability, which is heavily influenced by the recovery of schools, hospitals, key retail, and financial services. Although our focus in this study is on hospital and school recovery, as influenced by their interaction and their interdependence with other physical systems, for a more comprehensive understanding of social services stability, future work should focus on the integration of other resilience metrics (e.g., retail and financial services). Furthermore, a more holistic understanding of community resilience might require further integration of all relevant resilience metrics that correspond to the five mentioned resilience goals^48^. Resilience is defined graphically in this study as the area underneath the functionality curve^47^, ℱ, from the hazard occurrence time, *t*_0_, to the full recovery time, *TR*, as follows:

4 $$R = \mathop \smallint \limits_{t0}^{TR} \frac{{\mathcal{F}}\left( t \right)}{TR}dt$$

#### Community’s social services stability

Providing appropriate medical and education services is critical for the community. In this study, the social services stability index (*SSSI*) is introduced as another measure of community strength after disasters, and it measures the accessibility of community individuals to the main public services with a focus on healthcare and education as pivotal services after disasters. The *SSSI* is constructed as a composite indicator in which the wide-spread additive aggregation method, called the summation of weighted and normalized indicators method^78^, is utilized as follows:

5 $$SSI\left( t \right) = \mathop \sum \limits_{i = 1}^{N} w_{i} \left( t \right)\left( {\frac{{\mathcal{F}}_{i} \left( t \right)}{{\mathcal{F}}_{i,ult} }} \right) \quad \forall t = t_{0} , \ldots , TR$$

where $w_{i}$ and *ℱ*_*i*_ are the weighting factor and functionality for service *i*, respectively, at time *t* ranged from the disaster occurrence time, *t*_0_, to the full recovery time, *TR*.

Each resident's need for these services is different. For instance, the number of hospital visits for seniors is significantly higher than any other age group^79,80^, and residents that do not have schoolchildren are not concerned with education availability. Therefore, the weighting factor *w*_*i*_ spatially simulates these varying needs for the healthcare system by incorporating the expected average number of hospital visits for each family. On the other hand, the number of schoolchildren per family is used to predict the *w*_*i*_ for this family’s education service. *w*_*i*_ is also temporally modeled to mimic the community changing demand overtime after the disaster. A higher value of *w*_*i*_ is assigned to the healthcare system immediately after the disaster and until the hospitals’ demand returns to normalcy. In contrast, a minimal value of *w*_*i*_ is given to the education system during the school recess.

## Supplementary Information

Supplementary Information.

## Data Availability

All data used in the analysis are included in the paper and the SI. Additional data related to this paper may be requested from the corresponding authors.

## Code availability

The agent-based model components have been described in the paper and the SI and are made available from the corresponding author on request.
