# Supplementary material for: Healthcare and education networks interaction as an indicator of social services stability following natural disasters
Source: Sci Rep. 2021 Jan 18;11:1664. doi: 10.1038/s41598-021-81130-w (PMC7814048; doi:10.1038/s41598-021-81130-w)
Supplement: Supplementary file 1 — Supplementary Information. [file 41598_2021_81130_MOESM1_ESM.pdf]

Supplementary Information: Healthcare and Education Networks Interaction as an Indicator of Social Services Stability Following Natural Disasters

Emad M. Hassan, Hussam Mahmoud*****

Department of Civil and Environmental Engineering, Colorado State University, Fort Collins, CO, USA
Email: Hussam.Mahmoud@colostate.edu
Tel: 970-491-6605

The presented agent-based model for the healthcare and education systems comprises three different levels a) systems level including healthcare and education networks, b) agents level comprising hospitals, schools, school districts, and the supporting infrastructure, buildings, and suppliers, and c) sub-agent level containing all individuals within the community that are either staff or receptor of medical or education services or neither of the two. The interaction topology used to define the relationships within each agent as well as between different agents is also included in the analysis. The model simulates the decision-making process and the ability of each system, agent, or sub-agent to mitigate and recover after a disaster. The agent type, attributes, and decision domain are summarized in **Error! Reference source not found.**. More details about the model components are discussed below.

Table S1: Components of the presented agent-based model.

Type	Agent type	Attributes	Decision making
Systems	Healthcare	Comprises hospitals and all their sub-components including buildings, infrastructure, suppliers, and staff.	Controls all medical services in the community. Aggregates all the decisions made by its components (agents).
	Education	Incorporates school districts, schools and all their sub-components including infrastructure, suppliers, and staff.	Controls all the education services in the community. Aggregates all the decisions made by its components (agents).
Main agents	Hospital	Provides medical services for all patient's categories. Depends on staff, utilities, space, and supplies.	Makes all decisions related to the medical services by aggregating the decisions made by its sub-components.
	School	Provides education services for students at a specific grade. Depends on staff, utilities, space, and supplies.	Makes all decisions related to the education services by aggregating the decisions made by its sub-components.
	School district	Refers to the local administration of schools and depends on staff, utilities, space, and supplies.	Regulates, manages, and monitors the education services for all the schools that belong to the district. Allocates resources for all the schools within the district.
Supporting Agents	Building	Refers to a housing unit in the community and can have different archetypes, structural system, damage probability, number of residents, etc.	Provides shelter for community individuals. Can collapse during the earthquake causing casualties.
	Water	Represents the municipal water network and includes different components such as water treatment plants, pumps, storage units, and distribution pipelines.	Responsible for operating and maintaining municipal water networks. Controls the repair and recovery process of the water network. Some hospitals and schools also operate their water tank.
	Power	Exemplifies the electricity network and includes different components	Responsible for operating and maintaining municipal power networks. Controls the repair

		such as stations, substations, distributing circuits, and distribution lines.	and recovery process of the power network. Some hospitals and schools also operate their emergency power generators.
	Transportation	Describes the transportation network and includes roads and railway systems.	Responsible for operating and maintaining the transportation networks, including those used to reach hospitals and schools. Controls the repair and recovery process of the transportation network. Can provide detours to avoid damaged roads.
	Telecommunication	Refers to either cell phones, landlines, or other networks.	Responsible for operating and maintaining telecommunication networks. Controls the repair and recovery process of the telecommunication network. Some hospitals and schools also operate their internal network.
	Wastewater	Refers to the sewer network and includes collecting lines, pumps, and treatment plants.	Responsible for the collection of wastewaters. Controls the repair and recovery process of the wastewater network. Some hospitals and schools are provided with a backup system to collect and process wastewater.
	Natural gas	Refers to the fuel network and used for heating and other purposes in hospitals and schools and its components include stations and distributing pipelines.	Responsible for the delivery of natural gas. Controls the repair and recovery process of the natural gas network. Some hospitals and schools can store natural gas to use in emergency cases.
	Medical supplies	Includes oxygen, surgical, and Rx suppliers for hospitals.	Controls the delivery of medical supplies to hospitals.
	Non-medical supplies	Comprises of Food, book, and technology suppliers for hospitals and schools.	Controls the delivery of basic supplies to hospitals and schools.
Sub-agents	Hospital staff	Denotes an individual employed by a hospital. Subcategorized into physicians, nurses, supporting staff, and alternative staff.	Can decide to work for another hospital, reduce the patient treatment time, transfer the patients, work additional time to cover staff shortage, etc.
	School staff	Refers to an individual employed by a school or school district. Subcategorized into teachers, supporting staff, volunteers, and administrative staff.	Can decide to work for another school, control and monitor the student outcomes based on their experience, control the admission process (if they are in admin staff), work additional time to cover the staff shortage, teach different grades or topics if needed, etc.
	Patient	Implies any person receiving medical treatment in the hospital. Subcategorized into normal and earthquake-related patients.	Can decide to select the proper hospital (only if his/her case is not critical), accept or refuse the transfer, use an ambulance or private transportation to go to the hospital, pay for medical services if not insured, evaluate the provided services, etc.
	School student	Refers to schoolchild and can be in any grade between kindergarten and grade 12.	Can decide to go to school, select the school during the admission stage (school of choice application), use school transportation or private transportation, etc.
	Student guardian	Refers to a student's parent or, in some cases, another individual responsible for the student and in this case he/she must share the home with the student.	Can decide to send their schoolchildren to school, select the school during the admission stage (school of choice application), choose school transportation or private transportation for the student, monitor student outcomes, be responsible for providing homeschooling if needed, etc.
	Another individual	Any community individual who is not mentioned above.	Can decide to be a volunteer in healthcare or education facilities. Can share their home with individuals related to the hospitals or schools and impact their decisions.

1. Hospitals' functionality estimation

To quantify the capacity of healthcare facilities, the success tree in Fig. S1 is utilized. The availability condition of each sub-component (basic event) at time t is calculated to estimate the probability of staffed bed availability, P_B, which is used to determine the total available number of staffed beds at each healthcare facility. The mean value of P_B represents the quantity functionality, Q_V, of this healthcare, which is calculated as: $E[Q_V(t)] = (\frac{1}{N_{em}}\sum_{n=1}^{N_{em}} P_B^n)^{\alpha_{em}} + (\frac{1}{N_{in}}\sum_{n=1}^{N_{in}} P_B^n)^{\alpha_{in}}$. Where, N_{em} and N_{in} are the number of emergency and inpatient beds in the investigated facility, respectively, α_{em} and α_{in} are weighting factors for emergency and inpatient beds, respectively, and B is the total number of the staffed beds.

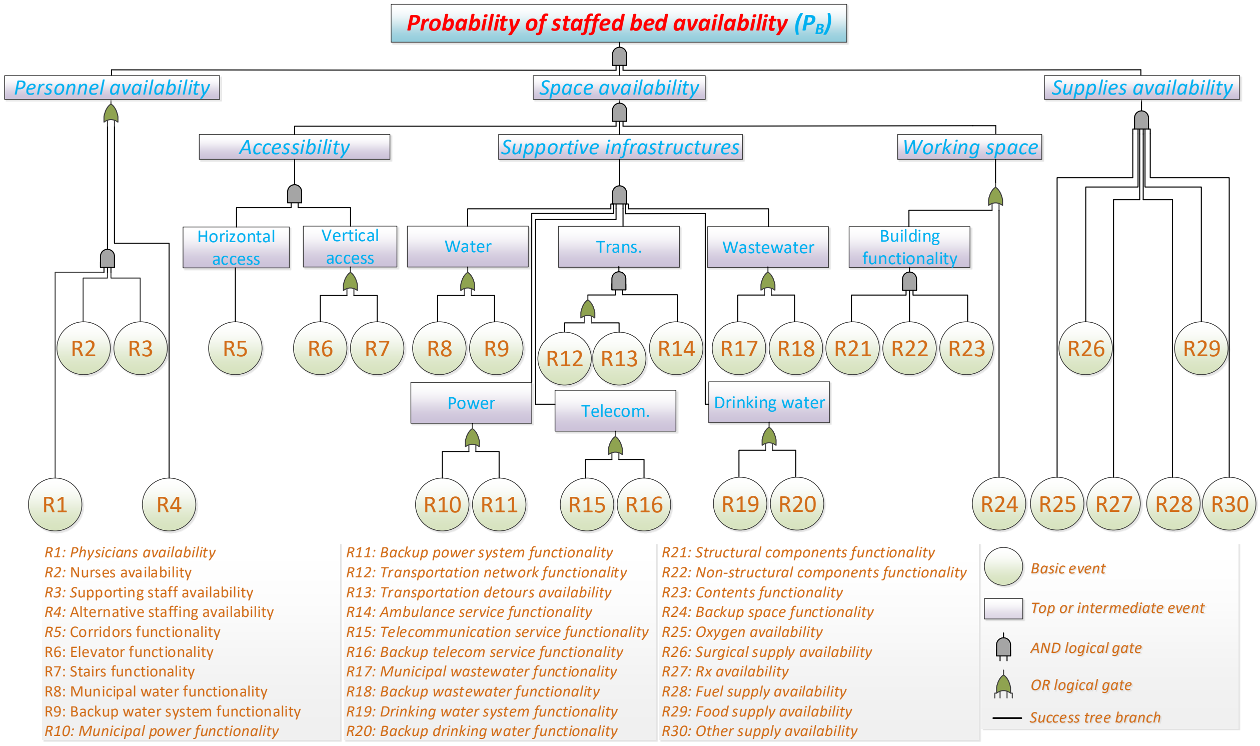

Fig. S1. Success tree for determining the availability of staffed beds in a hospital.

In this study, we evaluated the basic events based on the temporal states of different agents, supporting agents, and sub-agents. Even though this method is computationally expensive, it is a practical method to estimate these basic events. To model the effect of the sub-agents that directly or indirectly impact the education system on the hospital's functionality, the personnel availability events (i.e. sub-components *R1* to *R4*) are aggregated from the sub-agents' availability to work. This allows the model to count each staff availability separately and consider the staff as absent for the following cases: a) the employee is hospitalized or cannot find an available bed, b) the employee has a family member who needs hospitalization service and could not find an available bed, c) the employee has a schoolchild who is homeschooled and he/she is the only guardian, and d) the employee has a schoolchild who missed school for more than one semester (employee is assumed to out-migrate). Similar to the sub-agents, the availability of utilities and supplies are evaluated as supporting agents so that the effect of the shared utilities and suppliers on hospitals and schools is modeled.

Accessibility to medical services, S_A, is calculated, per Eq. S1, as a function of the patient waiting time, W_t, before being seen by medical staff, which is estimated using Eq. S2.

$$S_A = [W_t^{max} - W_t^{a})]/[W_t^{max} - W_t^{b}] \geq 0.0 \quad \text{(S1)}$$

$$W_t^a = W_0 + T_{tvl} + a_t[B_0 - B_t]/B_0 + a_e[N_t - N_0]/N_0 \quad \text{(S2)}$$

where, W_0 is the basic waiting time, T_{tvl} is the patient travel time, a_t is the effect of the reduction in the staffed beds, and a_e is the effect of the increase in the total patients' number. The ratio between the patient waiting time after, W_t^a, and before, W_t^b, the disaster is used while considering the maximum allowable waiting time, W_t^{max}.

To estimate the effectiveness of the offered medical service, S_E, the patient treatment time, T_t, is utilized as shown Eq. S3. T_t can change after disasters because of the higher demand on healthcare facilities and may significantly reduce the patient outcomes. T_t is determined, at any time t, as $T_t^t = f(R1^t/N_n^t, PCC^t)$. Where $R1$ is the available physician, N_n is the current hospital demand, and PCC is the patient case criticality. To measure the healthcare effectiveness, the ratio between the patient treatment time after, T_t^a, and before, T_t^b, the earthquake is used, while considering the minimum allowable treatment time, T_t^{min}.

$$S_E = [T_t^a - T_t^{min})]/[T_t^b - T_t^{min}] \geq 0.0 \quad \text{(S3)}$$

2. Schools' functionality estimation

Schools' enrollment capacity is quantified using the success tree in Fig. S2. The quantity functionality of the educational services, S_V, is given by the expected number of available seats for students at grade i in each school as $E[S_V(\mathrm{t})] = \sum_{i=i}^{I_g} \frac{1}{N_i} \sum_{n=1}^{N_i} P_{s,i}^n$. Where, N_i is the total number of seats at grade i and I_g is the number of grades.

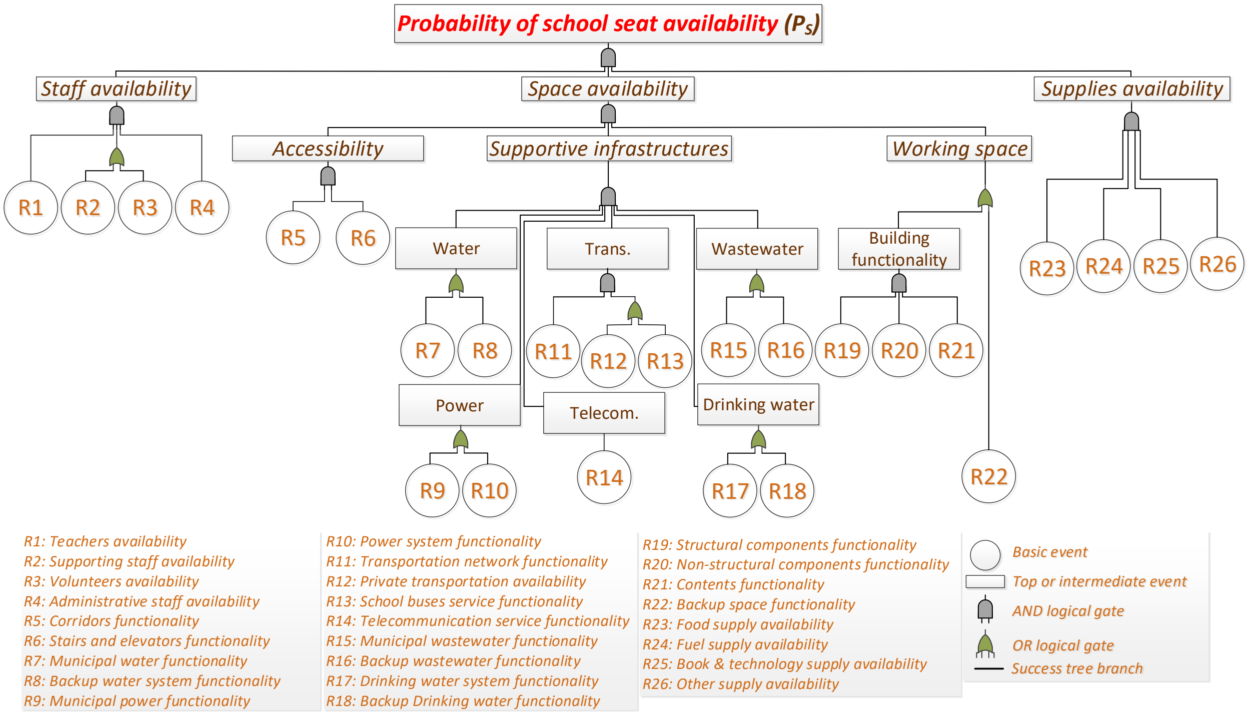

Fig. S2. Success tree for determining the availability of seats in a school.

Similar to the staffed beds' quantification, we utilized the temporal states of different agents, supporting agents, and sub-agents to evaluate the main events in this success tree. Specifically, the effect of sub-agents that have a direct or indirect relationship with the healthcare system on the

school's functionality, personnel availability events, sub-components from R1 to R4, are aggregated from the sub-agent's availability to work. The school's staff is considered absent for the previously mentioned cases. The availability of the utilities and supplies are evaluated as supporting agents.

The quality of the educational services, S_S, is calculated, as shown in Eq. S4, as combination of various quality measures including teacher assignment, T_a, and experience, T_e, as indicators of teacher quality*;* class size, C_s, and technology, C_t, as indicators of classroom quality*;* and leadership, S_l, and professional community, S_{pc}, as indicators of school quality.

$$S_S(t) = \{T_a^{\alpha_a}(t)T_e^{\alpha_e}(t)\}\{C_s^{\alpha_s}(t)C_t^{\alpha_t}(t)\}\{S_l^{\alpha_l}(t)S_{pc}^{\alpha_{pc}}(t)\} \tag{S4}$$

Where, the α-terms are weighting factors that represent the importance of each quality measure. Teacher assignment is evaluated using Eq. S5.

$$T_a(t) = \sum_i \left\{\frac{ST_{i,req}(t) - E\left(ST_{i,unq}(t)\right)}{ST_{i,req}(t)}\right\} \tag{S5}$$

Where, the required staff, $ST_{i,req}$, varies with the number of students enrolled, $N_i(t)$, and the class capacity, $R_i(t)$ as follows $ST_{i,req}(t) = \frac{N_i(t)}{R_i(t)}$. The probability of finding alternative staff, $P(ST_{alt})$, is $P(ST_{i,alt}(t)) = P(ST_i < ST_{i,req}|ST_{avl})$, which is a function of the required staff and available staff to hire, ST_{avl}. However, it is probable that this replacement staff may be less qualified, $P(ST_{unq})$ than the permanent staff, which is calculated as $P(ST_{unq}(t)) = P(ST_{i,alt})P(ST_{mis}|ST_{i,alt})$.

Teacher experience is calculated, using Eq. S6, as the ratio between the experienced staff and the total staff, $ST_i(t)$ as follows:

$$T_e(t) = \sum_i \left\{\frac{ST_i(t) - E\left(ST_{i,inexp}(t)\right)}{ST_i(t)}\right\} \tag{S6}$$

where the expected value of inexperienced teachers is calculated as $P\left(ST_{inexp}(t)\right) = P\left(ST_{i,add} \cap ST_{mis}\right) = P\left(ST_{i,add}\right)P\left(ST_{mis}|ST_{i,add}\right)$ as a function of the conditional probability of a new teacher joining the school staff, ST_{add}, and does not have enough teaching experience, ST_{mis}. The classroom size is measured as a ratio between existing, $R(t)$, and normal, $R(0)$, teacher-to-student ratios, considering maximum acceptable class capacity, R_{max} as shown in Eq. S7. The mean value of the classroom sizes for all grades is utilized to express the overall school quality as follows $C_s(t) = \overline{C_{\iota,s}}(t) \quad \forall i$.

$$C_{i,s}(t) = \frac{R_{i,max} - R_i(t))}{R_{i,max} - R_i(0)} \leq 1.0 \tag{S7}$$

The ratio between the current technology availability, $T(t)$, and the technology before disaster occurrence, $T(0)$, is used as an index for classroom technology, as shown in Eq. S8. This technology availability is calculated as a function of building contents damage, L_C, and deficiencies in essential utilities, U, for classroom technology, such as power and telecommunication as follows $P\left(T_i(t)\right) = P\left((1 - \left(L_{i,C}\right))\cap U_i\right)$.

$$C_t(t) = 1 - \sum_i \left\{\frac{T_i(0) - E\left(T_i(t)\right)}{T_i(0)}\right\} \tag{S8}$$

The leadership availability is modeled as a function of current leadership availability, $l(t)$, following the disaster as well as at time $l(0)$ before disaster occurrence as shown in Eq. S9. The quality of

leadership is estimated as a function of the school administration, ST_{admin}, and experienced faculty, ST_{lp}, as $P(l_i(t)) = P(ST_{admin} \cup ST_{lp})$.

$$S_l(t) = 1 - \sum_i \left\{\frac{l_i(0) - E(l_i(t))}{l_i(0)}\right\} \quad \text{(S9)}$$

The professional community's availability is estimated as the ratio between the current professional community, $p_c(t)$, and its value before the disaster, $p_c(0)$, as shown in Eq. S10. Appointing unqualified teachers, ST_{unq}, and the teacher change events, ST_{ch}, are utilized as an indicator of the professional community as follows $P(pc_i(t)) = P\{(1 - ST_{unq}) \cup (1 -)\}$.

$$S_{pc}(t) = 1 - \sum_i \left\{\frac{pc_i(0) - E(pc_i(t))}{pc_i(0)}\right\} \quad \text{(S10)}$$

3. Decision-making models

Fig. S3 shows the methodology used in this study to model the main agent (hospital and school) drop of functionality after natural hazards and the required decisions to return to normalcy.

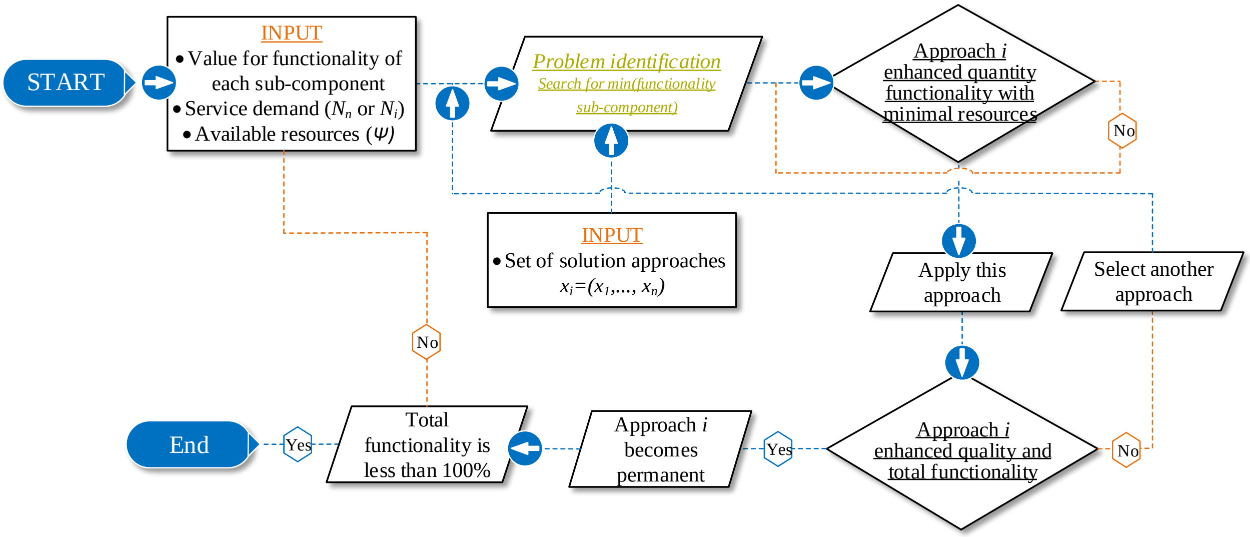

Fig. S3. Decision-making algorithms.

Patient-driven model

The patient-driven model is used to estimate the patient demand on each healthcare facility (hospital). This model calculates the probability $p_{i,n}$ of a patient i going to a healthcare facility n using different factors that affect the selection of a healthcare facility, as shown in Fig. S4.

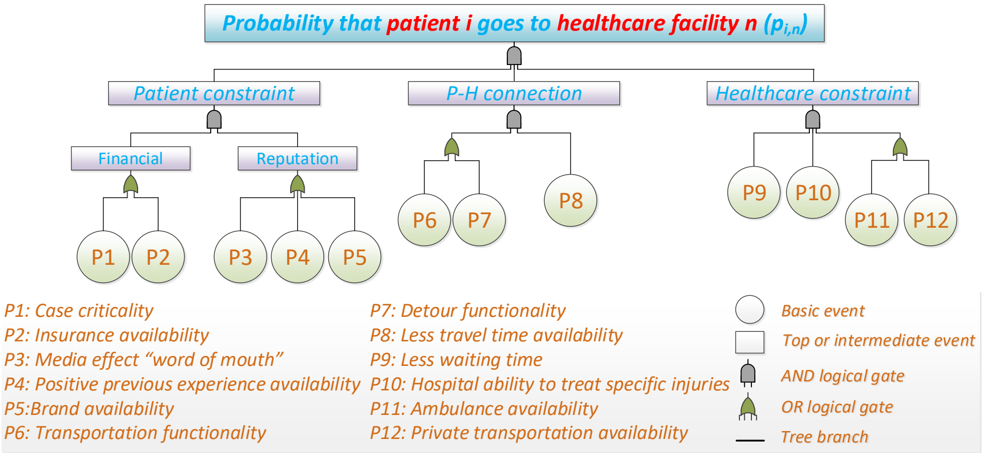

Fig. S4. Patient-driven model probability.

The patient selection probability vector $\boldsymbol{P_p}$ is built for all the healthcare facilities in the investigated community, N, and is described as $\boldsymbol{P_p(t)} = \left[p_{i,1}\ p_{i,2}\ p_{i,3} \cdots\ p_{i,N}\right] = p_{i,n}$. The healthcare facility with the highest probability is considered as the one selected by the patient, $\lambda_{i,n}$ as illustrated in Eq. S11. Assuming that the community has a total number of patients, N_t, then the expected number of patients, N_n, at a facility, n, can be estimated as the expected total number of patients who will select this hospital and can be represented as $E[N_n(t)] = \sum_{i=1}^{N_t} \lambda_{i,n}$. The expected demand for healthcare facilities might change further due to the patient transfer process, which will be discussed in the next section.

$$\lambda_{i,n} = \begin{cases} 1.0 & \Leftrightarrow \left(Max_{n\epsilon[1:N]} p_{i,n} - p_{i,n} = 0.0\right) \\ 0.0 & \Leftrightarrow \left(Max_{n\epsilon[1:N]} p_{i,n} - p_{i,n} \neq 0.0\right) \end{cases} \qquad \text{(S11)}$$

Healthcare facilities interaction model

The interaction between healthcare facilities is considered in the redistribution of services, repair resources, medical staff, and patients. The probability, $p_{m,n}$, of patient transfer from a healthcare facility m to facility n is calculated using the probability tree shown in Fig. S5.

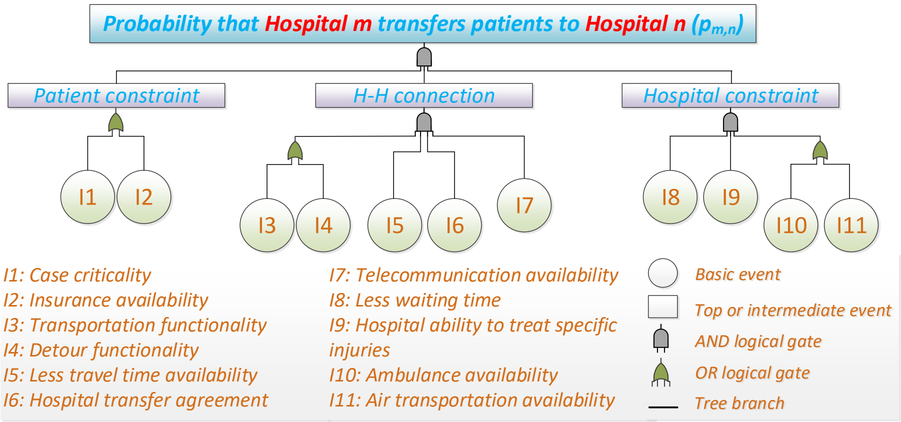

Fig. S5. Hospital interaction probability.

The total number of patients transferred, $N_{dist\ m \to n}$, from hospital m to hospital n, is calculated based on Eq. S12.

$$N_{dist_{m\to n}}(t) = \left(N_m(t) - \varepsilon_m(t)\right)\frac{p_{m,n}(t)}{\sum_{k=1}^{N}\{p_{m,k}(t)\}} \ \ \forall\, n,m \qquad \text{(S12)}$$

Where, $\varepsilon_m(t)$ is the maximum capacity of the hospital *m*, $p_{m,n}(t)$ is the interaction value between hospital *m* and hospital *n*, and *N* is the number of hospitals that can receive the transferred patient.

Healthcare facilities transfer staff to close the staff shortage gap in other facilities. To estimate the number of additional staff, $M_{s\ m\to n}$, transferring from hospital *m* to hospital *n*, Eq. S13 is used

$$M_{s_{m\to n}}(t) = \begin{cases} \left(ST_{req_n}(t) - ST_n(t)\right) * \frac{p_{ST_{m,n}}(t)}{\sum_{k=1}^{N}\{p_{ST_{m,k}}(t)\}} \Leftrightarrow ST_n < \min(SP_n, SU_n) \\ 0.0 \Leftrightarrow ST_n \geq \min(SP_n, SU_n) \end{cases} \ \ \forall\, n,m \qquad \text{(S13)}$$

$$p_{ST_{m,n}}(\text{t}) = p\left(E_{ag_{m,n}} \cap E_{tf_{m,n}} \cap E_{ma_{m,n}} \middle| ST_m > \min(SP_m, SU_m)\right)$$

Where, ST_{req_n} is the required number of staff at hospital *n* and p_{ST} is the probability of the staff transfer. This probability is calculated based on the expected values of agreement availability, E_{ag}, between hospital *m* and *n*, the hospital *m* staff accepting a transfer, E_{tf}, and the transferred staff will match the need of hospital *n*, E_{ma}. However, this transfer will occur if and only if hospital *m* has more staff than required.

Healthcare facilities can also transfer supplies as shown in Eq. S14 to determine the number of supplies, $SU_{add\ m\to n}$, transferred from hospital *m* to hospital *n*.

$$SU_{add_{m\to n}}(t) = \begin{cases} \left(SU_{req_n}(t) - SU_n(t)\right) * \frac{p_{SU_{m,n}}(t)}{\sum_{k=1}^{N}\{p_{SU_{m,k}}(t)\}} \Leftrightarrow SU_n < \min(ST_n, SP_n) \\ 0.0 \Leftrightarrow SU_n \geq \min(ST_n, SP_n) \end{cases} \ \ \forall\, n,m \qquad \text{(S14)}$$

Where,

$$p_{SU_{m,n}}(\text{t}) = p\left(E_{ag_{m,n}} \cap E_{tr_{m,n}} \cap E_{ma_{m,n}} \middle| SU_m > \min(ST_m, SP_m)\right)$$

Where, SU_{req} is the required amount of supplies at hospital *n and* p_{SU} is the probability of supplies transfer. Values of p_{SU} depend on the established agreement (E_{ag}), transportation functionality between hospital *m* and hospital *n* (E_{tr}), and the supplies matching the hospital needs (E_{ma}). However, this transfer will occur if and only if hospital *m* has more supplies than required.

School administration model

Fig. S6 shows the framework for students' admission and transfer.

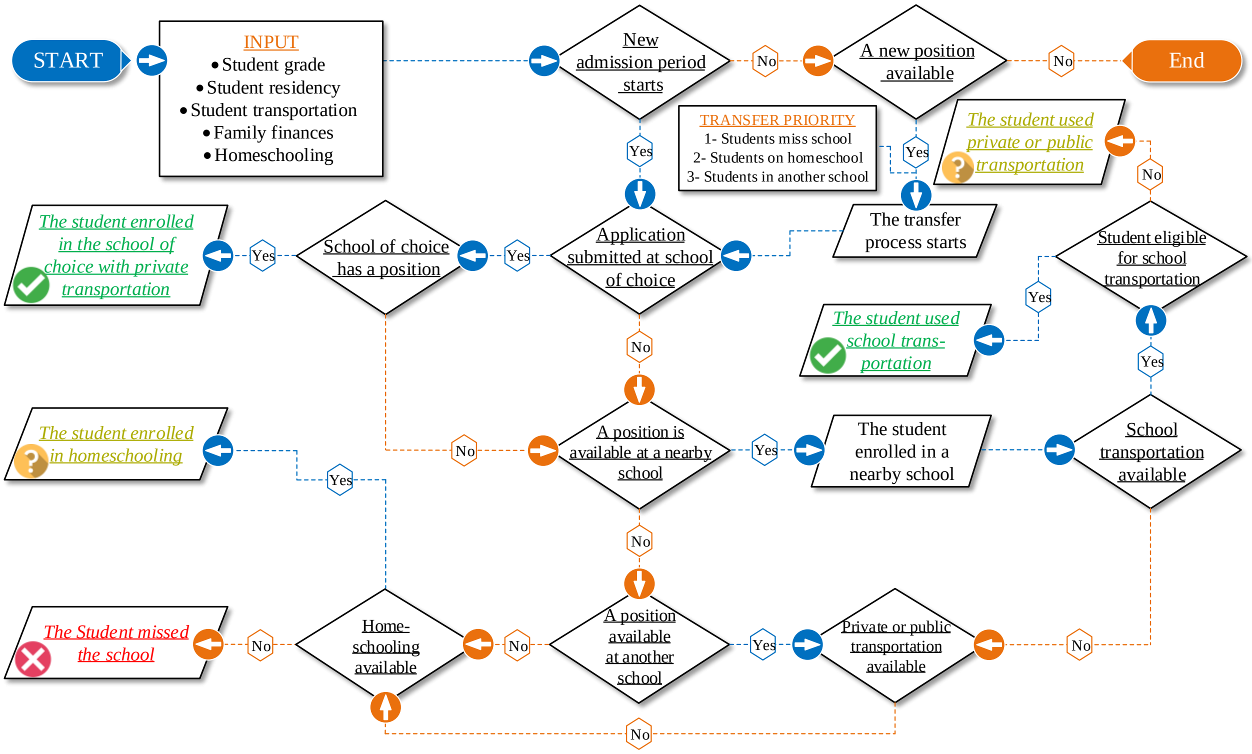

Fig. S6. Students' enrollment and transfer process framework.

Reopening damaged schools after major disasters involves the school district, school administrators, the building and fire departments, office of public safety, and the community. Among different approaches, the presented model considers that schools can be partially opened using backup spaces during the recovery stage to provide education for a limited number of students.

Schools can appoint staff in different categories, including temporary, part-time and permanent. This staff can also be transferred between schools to close the gap in staff shortage as shown in Eq. S15.

$$ST_{add}(t)_n = \begin{cases} \left(ST_{req_n}(t) - ST_n(t)\right) P(ST_{ap_n}(t)) \Leftrightarrow ST_n \leq \min(SP_n, SU_n) \;\&\; \sum ST_{req} \geq \sum ST \\ \left(ST_{req}(t)_n - ST_n(t)\right) P(ST_{tr_{m\to n}}(t)) \Leftrightarrow ST_n \leq \min(SP_n, SU_n) \;\&\; \sum ST_{req} < \sum ST \\ 0.0 \Leftrightarrow ST_n > \min(SP_n, SU_n) \end{cases} \quad \text{(S15)}$$

Where, E_{hr} is the availability of human resources, E_{ma} is the staff matching the school needs, and E_{fu} is the funding availability.

The appointment or transfer of staff can be calculated per Eq. S16 as:

$$P(ST_{tr_{m\to n}}(\mathrm{t})) = P\left(E_{w_{m,n}} \cap E_{tf_{m,n}} \cap E_{ma_{m,n}} \middle| ST_m > \min(SP_m, SU_m)\right) \quad \text{(S16)}$$

Where, appointment or transfer is assumed to take place only when space and supplies are sufficient to accommodate additional staff and the existing staff, ST, are less than the required, ST_{req}. The probability of staff transfer between school m and n is calculated based on the willingness of the school district to transfer the staff between schools, E_w, the school m staff accepting a transfer, E_{tf}, and the transferred staff will match the need of school n, E_{ma}.

Schools can also transfer supplies and resources to reduce the impact of the earthquake consequences on the school system as shown in Eq. S17.

$$SU_{add}(t)_n = \begin{cases} \left(SU_{req}(t)_n - SU_n(t)\right) P(SU_{tr_{m\to n}}(t)) \Leftrightarrow & SU_n \le \min(ST_n, SP_n) \\ 0.0 \Leftrightarrow & SU_n > \min(ST_n, SP_n) \end{cases} \tag{S17}$$

Where,

$$P(SU_{tr_{m\to n}}(\mathrm{t})) = P\left(E_{ag_{m,n}} \cap E_{tf_{m,n}} \cap E_{ma_{m,n}} \middle| SU_m > \min(ST_m, SP_m)\right) \tag{S18}$$

Where, E_{ag} is the established agreement, E_{tr}, is the availability of transportation, and E_{ma} is the supplies matching the school need.

Communities can support education through supporting the school staff, providing donations, and encouraging students and staff to keep the school system as functional as possible. To estimate the total number of volunteers at each school, the probability that a citizen responds to a request from the school is modeled using the community demographic data, including gender, age, education, and income of the volunteers.

4. Resilience quantification model

The introduced framework to investigate total healthcare and education system resilience is shown in Fig. S7.

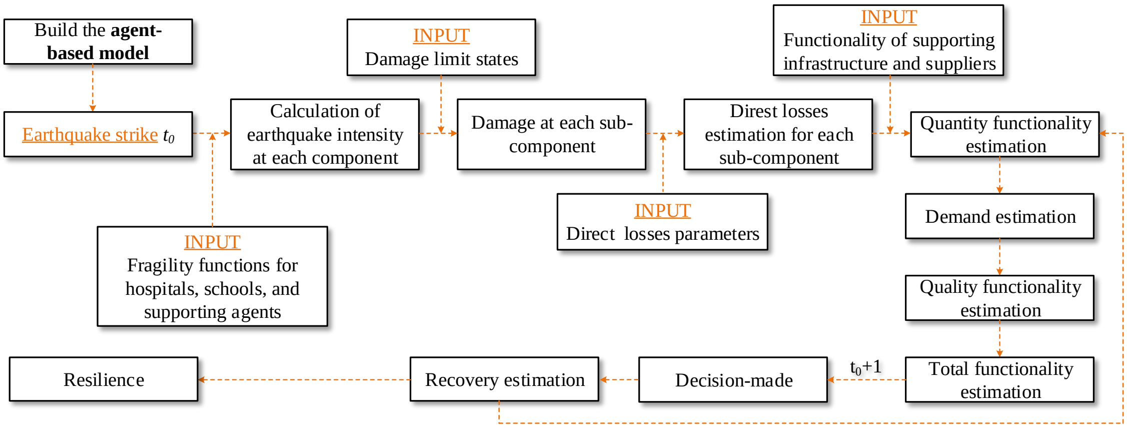

Fig. S7. Healthcare and education system resilience quantification approach.

Repair or restoration of each facility is estimated using a semi-Markov chain process, in which the restoration process is defined by discrete nondecreasing states. In the Markov chain process herein, the repair process at any time step can either improve the restoration state or not effect it. The current restoration state depends on the previous state but is independent of other previous states. The facility's quantity functionality is subcategorized into sub-components based on the repair crew specialty: structural components, building envelope, permanent and moveable partitions, mechanical equipment, and electrical systems. The discrete Markov chain modeling the recovery process is shown in Eq. S19.

$$Q_l(k\Delta t) = Q_l(0) \prod_{i=0}^{k-1} A_l \, \boldsymbol{P_t}(\boldsymbol{i}\Delta \boldsymbol{t})_{\boldsymbol{l}} \tag{S19}$$

Where, the functionality of sub-component l, Q_l, after time $k\Delta t$ is assumed to be related to the initial functionality drop, $Q_l(0)$, due to seismic damage, the interaction between the repair process of each facility and other community lifelines, A_l:

$$A_l = \prod_{j=1}^{N_f} \beta_j \tag{S20}$$

The interaction term A_l is calculated based on factor β_j, defined in terms of the interaction factor, e_j, and the current functionality state of the lifeline j, $Q_j(t)$.

$$\beta_j(t) = \begin{cases} 1.0 \Leftrightarrow & e_j = 0.0 \\ Q_j(t)/e_j \Leftrightarrow & 0.0 < e_j \leq 1.0 \end{cases} \tag{S21}$$

$$\boldsymbol{E} = [e_1 \quad e_2 \quad \cdots \quad e_{N_f}] = e_j \tag{S22}$$

The transition probability matrix, $\boldsymbol{P_t}$ is represented in Eq. S22 as:

$$\boldsymbol{P_t}(\boldsymbol{t}) = \begin{bmatrix} p_{1,1} & p_{1,2} & \cdots & p_{1,R-1} & p_{1,R} \\ p_{2,1} & p_{2,2} & & p_{2,R-1} & p_{2,R} \\ \vdots & & \ddots & \vdots & \\ p_{R-1,1} & p_{R-1,2} & \cdots & p_{R-1,R-1} & p_{R-1,R} \\ p_{R,1} & p_{1,2} & & p_{R,R-1} & p_{R,R} \end{bmatrix}_t \tag{S23}$$

Where, the probabilities $p_{s,r}(t)$ are defined in Eq. S24 as:

$$p_{s,r}(t) = Prob[Q(t) = Q_r | Q(t_0) = Q_s] \ , \ \sum_{s=1}^{R} p_{s,r}(t) = 1.0 \ \forall t \tag{S24}$$

The transition probabilities $p_{s,r}(t)$, shown in Eq. S25, are defined as the probability of the functionality state transitioning to the next (higher) level:

$$p_{s,r} = a\left[1 - e^{[-bx(0.1r^{0.5})]}\right] \tag{S25}$$

Where, a and b are parameters that refer to the geographical and structural properties of the investigated lifeline. The transition probabilities are also calculated as a function of the assigned repair crews, x, and the current restoration stage, r.

The repair crews are distributed by dynamic optimization to achieve the pre-defined community objectives regarding each service. Suppose that the total available number of repair crews at each specialty, m, is $X^m(t)$, which changes as a function of the time after the disaster. The decision makers assumed in this study assign these crews, $xm_n(t)$, at any time, t, to repair the damaged sub-components by crew's specialty, m, in each school, n, to achieve the maximum quantity of the offered education service for the whole community as denoted by Eq. S26.

$$\max_{\mathcal{F}_{V(t)}} \sum_{n=1}^{N} \mathcal{F}_V^n(t) \tag{S26}$$

Distribution of the repair crews is subjected to the following constraints a) limitation of repair resources, b) pre-defined repair sequence based on engineering judgment, and c) work environment constraint that limits the total number of repair crews, $x_{n,max}$, in any building, n, as a function of the building area, A_t, as shown in Eq. S27 and S28.

$$X^m(t) = \sum_{n=1}^{N} x_n^m(t) \tag{S27}$$

$$x_n \quad \leq \ x_{n,max} \qquad \forall x_{n,max} \ , \ x_{n,max} = 2.3 \times 10^{-6} A_t + 1.0, \ \ 2.0 \leq x_{n,max} \leq 26.0 \tag{S28}$$

5. Damage and recovery for the investigated community

The detailed damage for sub-components in healthcare and education facilities including structural, non-structural (drift sensitive and acceleration sensitive), and content sub-components and the direct social losses are shown in Fig. S8. The damage states (Fig. S9) and recovery trajectory (Fig. S10) for the supporting infrastructure.

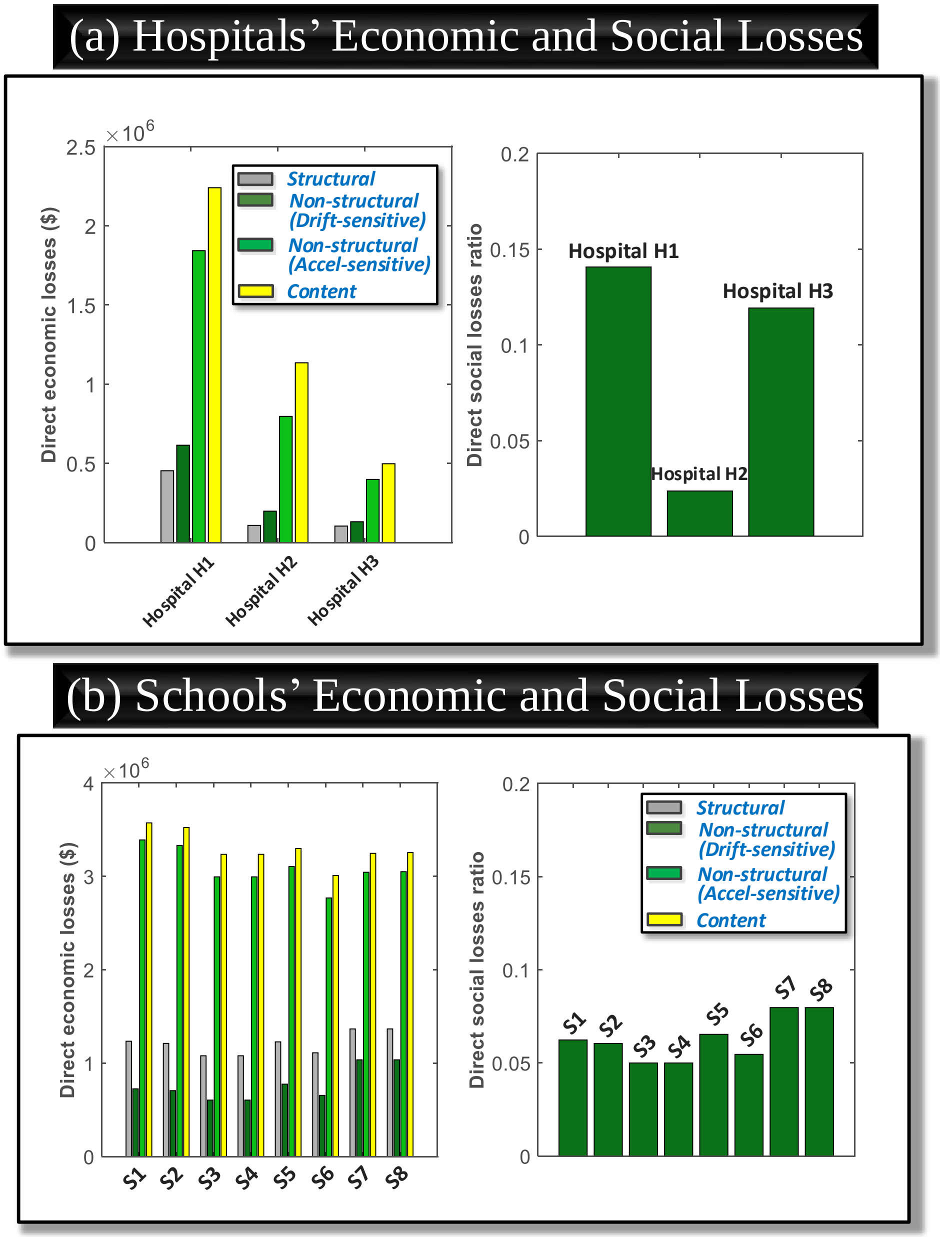

Fig. S8. Direct economic and social losses for a) hospitals and b) schools.

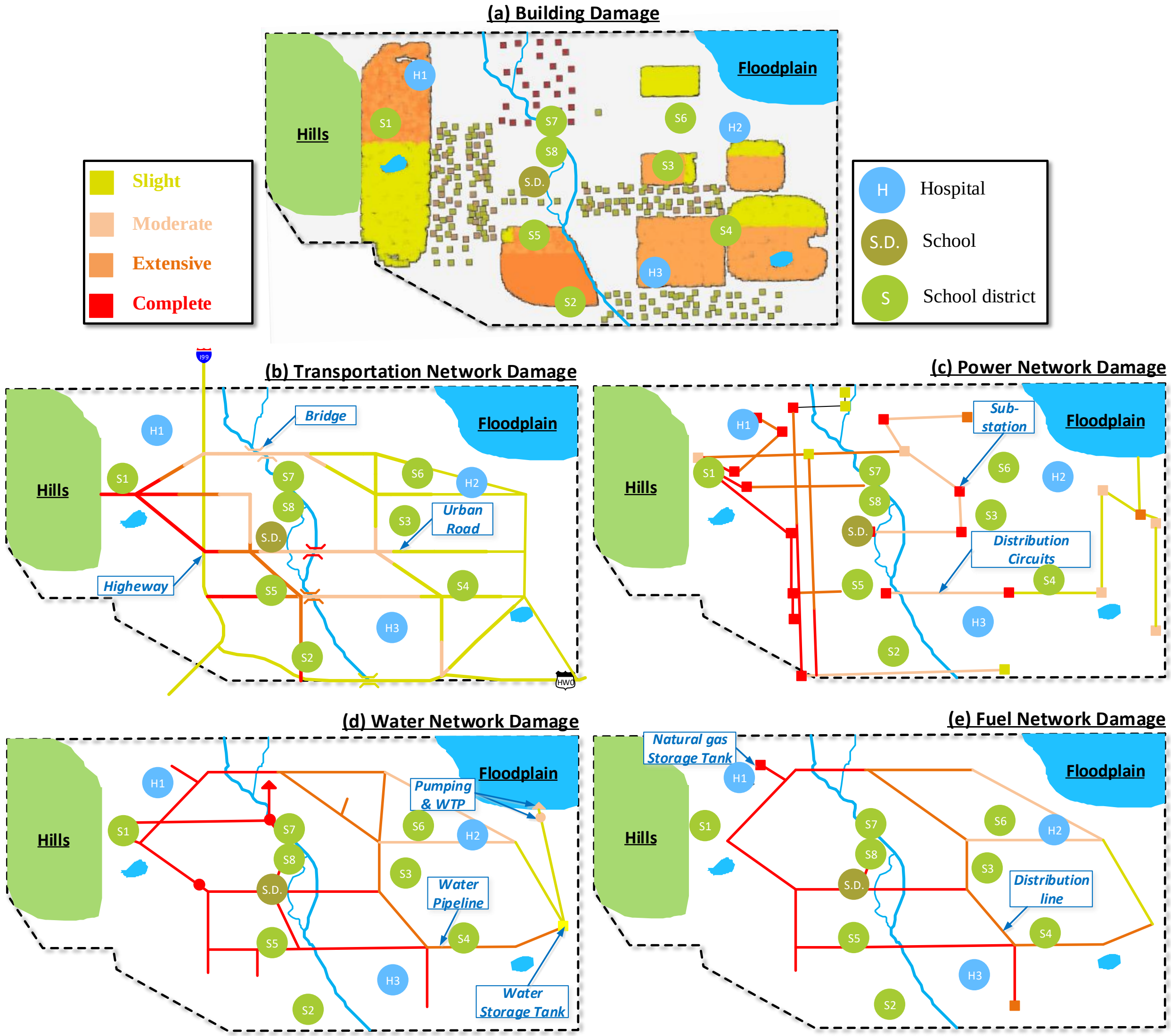

Fig. S9. Damage for the investigated community's-built environment including a) buildings, b) transportation network, c) power network, d) water network and e) fuel network.

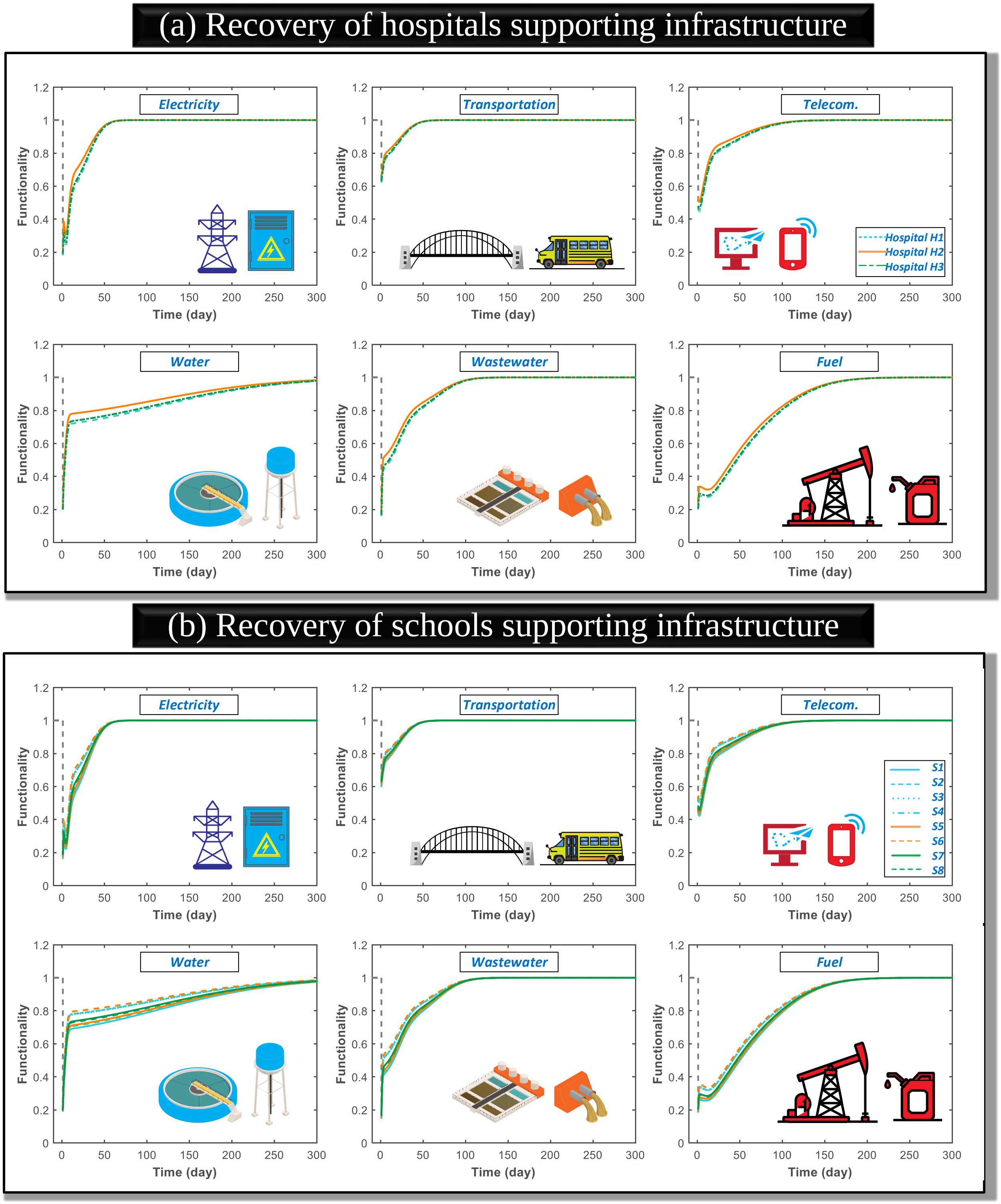

Fig. S10. Recovery trajectory for the supporting infrastructure for a) healthcare facilities and b) education facilities.
